# Genomic survey, characterization and expression profile analysis of the peptide transporter family in rice (*Oryza sativa *L.)

**DOI:** 10.1186/1471-2229-10-92

**Published:** 2010-05-20

**Authors:** Xiaobo Zhao, Jianyan Huang, Huihui Yu, Lei Wang, Weibo Xie

**Affiliations:** 1 National Key Laboratory of Crop Genetic Improvement and National Center of Plant Gene Research (Wuhan), Huazhong Agricultural University, Wuhan, 430070, China

## Abstract

**Background:**

Peptide transporter (PTR) family whose member can transport di-/tripeptides and nitrate is important for plant growth and development. Although the rice (*Oryza sativa *L.) genome has been sequenced for a few years, a genomic survey, characterization and expression profile analysis of the PTR family in this species has not been reported.

**Results:**

In this study, we report a comprehensive identification, characterization, phylogenetic and evolutionary analysis of 84 PTR family members in rice (OsPTR) as well as their whole-life expression patterns. Chromosomal distribution and sequence analysis indicate that nearly 70% of OsPTR members are involved in the tandem and segmental duplication events. It suggests that genome duplication might be a major mechanism for expansion of this family. Highly conserved motifs were identified in most of the OsPTR members. Meanwhile, expression profile of *OsPTR *genes has been analyzed by using Affymetrix rice microarray and real-time PCR in two elite hybrid rice parents, Minghui 63 and Zhenshan 97. Seven genes are found to exhibit either preferential or tissue-specific expression during different development stages of rice. Under phytohormone (NAA, GA3 and KT) and light/dark treatments, 14 and 17 *OsPTR *genes are differentially expressed respectively. *Ka/Ks *analysis of the paralogous *OsPTR *genes indicates that purifying selection plays an important role in function maintenance of this family.

**Conclusion:**

These investigations add to our understanding of the importance of OsPTR family members and provide useful reference for selecting candidate genes for functional validation studies of this family in rice.

## Background

Nutrient transport is essential to life and occurs in both prokaryotes and eukaryotes. It is well established that peptide transporters play an important role in the nutrition of bacteria, yeasts and animals [1]. However, the role of transporters of small peptides (2-6 amino acids) in plants is less well defined [2]. Peptide transporter (PTR) family (*TC 2.A.17*), also called the proton-dependent oligopeptide transport (POT) family [3,4] is a family consists of di-/tripeptides transporters [5]. Besides, some plant PTRs acted as nitrate transporters are termed as the members of Nitrate Transporter 1 family (NRT1, [6]). PTR proteins in plants show significant sequence homology and contain several transmembrane (TM) regions, with a large hydrophilic loop among TMs. Generally, the identified PTR members in plants fall into three types based on the nature of their substrates: di-/tripeptides transporter, nitrate transporter and other substrates transporter [6].

AtPTR2 (At2g02040) was the first identified di-/tripeptides transporter in *Arabidopsis *[7-10] and had high mRNA expression levels in 3-d-germinating seed, root and young leaf [8]. The antisense plants of *AtPTR2 *exhibited delayed flowering time and arrested seed development [9,11]. AtPTR1 (At3g54140) transported di-/tripeptides with low selectivity as well as substrates lacking a peptide bond. *AtPTR1 *was expressed in the vascular tissue throughout the plant, indicative of a role in long-distance transport [10]. *AtPTR3 *(At5g46050) was a salt stress and mechanical wounding inducible gene, JA and SA were both involved in regulation of it [12,13]. AtPTR5 (At5g01180) which mediated high-affinity transport of dipeptides was most likely supplying peptides to maturating pollen, developing ovules and seeds. Overexpression of it resulted in enhanced shoot growth and increased N content [14]. The barley peptide transporter gene, *HvPTR1*, had seed-specific expression. Transport activity of HvPTR1 was regulated by phosphorylation in response to rising levels of amino acids in the germinating grain [15-17]. Functional di-/tripeptides transporters also have been reported in *Nepenthes *(NaNTR1, [18]), faba bean (VfPTR1, [19]) and *Hakea actites *(HaPTR4, [20]).

Although PTR is the abbreviation of peptide transporter, many cloned PTR members in plants are nitrate transporters, especially in *Arabidopsis*. AtNRT1.1 (At1g12110, CHL1), a dual-affinity nitrate transporter, was regulated by phosphorylation and involved in nascent organ development [21-26]. *AtNRT1.2 *(At1g69850, *NTL1*) encoded a low-affinity nitrate transporter. RNAi plants of it exhibited reduced nitrate-induced membrane depolarization and nitrate uptake activities [27]. The characterization of AtNRT1.4 (At2g26690) revealed the special role of petiole in nitrate homeostasis [28]. AtNRT1.5 (At1g32450) and AtNRT1.6 (At1g27080) were both plasma membrane proteins. *AtNRT1.5 *was expressed in root pericycle cells close to the xylem and participated in root xylem loading of nitrate [29] while *AtNRT1.6 *was only expressed in reproductive tissue and involved in delivering nitrate from maternal tissue to the developing embryo [30]. CsNitr1, a nitrite transporter in *Cucumis sativus*, had two isoforms: CsNitr1-S and CsNitr1-L. In contrast to that CsNitr1-S enhanced the efflux of nitrite from the cell, CsNitr1-L might load cytosolic nitrite into chloroplast stroma [31].

Amino acids and other substrates can also be transported by some PTRs. BnNRT1.2, isolated from *Brassica napus *could transport both nitrate and histidine [32]. AgDCAT1 localized at the symbiotic interface was a dicarboxylates transporter in *Alnus glutinosa *and the mRNA of *AgDCAT1 *was only detected in the nodules [33].

In rice, only two PTR genes have been functionally verified. OsNRT1 (LOC_Os03g13274), which displayed low-affinity nitrate transport activity was constitutively expressed in the most external layer of the root, epidermis and root hair [34]. *SP1 *(LOC_Os11g12740) which determined the panicle size had high expression level in the phloem of the branches of young panicles. Phylogenetic analysis implied that SP1 might be a nitrate transporter, however, neither nitrate nor other compounds transport activity could be obtained from it [35].

As the main staple food for a large segment of the world population, rice also serves as a model plant for monocotyledon species [36,37]. Nutrient transport is critical for plant growth and development, in which PTR family members may play important roles. This work focused on a comprehensive identification, characterization, phylogenetic and evolutionary analysis of all the PTR family members in rice (*OsPTR*) as well as their whole-life expression profiling. Moreover, it has been demonstrated that the expression of some *OsPTR *genes is regulated phytohormone (NAA, KT and GA3) and light/dark treatments.

## Results

### Collection and identification of PTR genes in rice

Domain search (PF00854) of MSU Rice Genome Annotation Project (MSU RGAP) database released 125 sequences, out of which 91 were unique. Keywords "PTR", "peptide transporter" and "proton-dependent oligopeptide transport" searching identified 58, 85 and 10 genes respectively. BLASTP and TBLASTN searches against rice genome in three databases: MSU RGAP, NCBI and KOME released 95, 85 and 116 sequences respectively. By removal of the same sequences and different transcripts of the same gene, we initially identified 93 putative PTR genes in rice. Protein sequences of these genes were subjected to Pfam and SMART searches for the presence of PTR domains. As a result, seven genes whose protein contained only partial PTR domain and two genes annotated as retrotransposons were excluded from further analysis. Taken together, a total of 84 genes were predicted to encode PTR proteins in rice. Eighty-three genes had corresponding locus IDs in MSU RGAP database and one gene (Os01g0960900) was only found in NCBI. For convenience, all the "LOC_" prefix of MSU RGAP locus IDs were omitted representing *OsPTR *genes or proteins for further analysis. Meanwhile, similar methods were used and 53 PTR family members in *Arabidopsis *(AtPTR) were identified. The detailed information about the full-length cDNA, transcript, chromosomal position, BAC accession, gene structure for each *OsPTR *gene and characteristics of corresponding proteins can be found in Additional file 1. Protein structures predicted by SMART are shown in Additional file 2. The putative transmembrane (TM) regions in each OsPTR protein are listed in Additional file 3.

### Chromosomal localization and gene duplication

**Figure 1:**
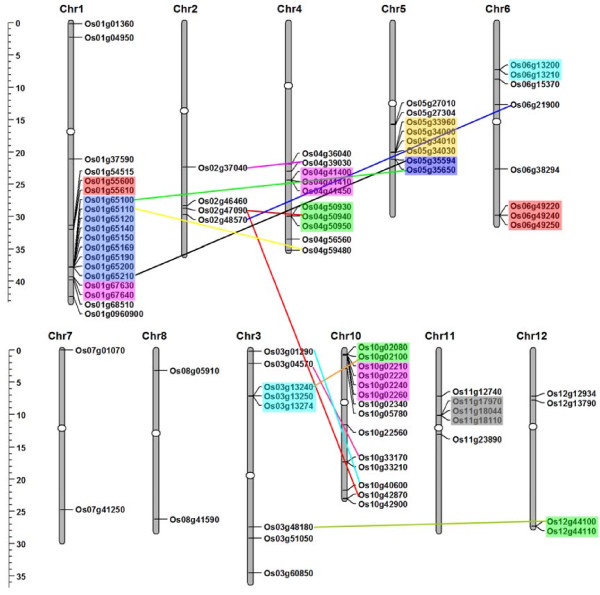
**Chromosomal localization and gene duplication events of *OsPTR *genes**. White ellipses on the chromosomes (vertical bars) indicate the position of centromeres. The scale on the left is in megabases (Mb). Respective chromosome numbers are indicated at the top of each bar. The chromosome order has been arranged to bring duplicated regions in the vicinity. The segmental duplication genes have been connected by straight line. In case of tandemly duplicated genes present in clusters, the genes are marked with rectangles of different colors.

To further investigate the relationship between the genetic divergence within the PTR family and gene duplication in rice, the chromosomal location of each PTR gene was determined from the genomic sequence of rice. The 84 *OsPTR *genes were dispersed on all the rice chromosomes except chromosome 9. Their chromosomal distribution pattern revealed that certain chromosomes and chromosomal regions had a relatively high density of *OsPTR *genes. For instance, chromosome 1 had the highest density with 19 members, followed by chromosome 10 had 14 members. Ten genes were located on chromosome 4, eight genes on chromosome 3, 5 and 6, five genes on chromosome 11 and four genes on chromosome 2 and 12, and only two genes on chromosome 7 and 8. For most chromosomes, *OsPTR *genes were present only on half arm of the chromosome or in clusters, i.e., no *OsPTR *gene appeared on the short arm of chromosome 2, 4 and 5 and most of the genes on chromosome 1 and 10 were clustered together (Figure 1).

Analysis of the MSU RGAP segmental duplication database revealed that 21 *OsPTR *genes (11 pairs) could be assigned to rice segmental duplication blocks (Figure 1). Os02g47090 participated in two segmental duplications with Os04g50940 and Os10g42870 respectively. The overall identities of the protein sequences of these genes ranged from 37.6% to 76.1%. A total of 44 genes (14 clusters, nine genes also involved in segmental duplication) were considered as tandem duplications according to the criterion adopted in our analysis, including the largest cluster of nine genes on chromosome 1, two four-gene clusters on chromosome 10 and 5, five three-gene clusters on chromosome 3, 4, 6 and 11, six two-gene clusters on chromosome 1, 5, 6, 10 and 12 (Figure 1). The protein homology of these genes varied from 22.9% to 89.5%. Interestingly, all the tandemly duplicated genes in the same cluster had the same direction of transcription except Os06g13200 and Os06g13210. This might suggest the conserved behavior of tandem duplications in this family. Of the 84 *OsPTR *genes, 66.7% (56 of 84) involved in the duplication events. Moreover, most of the duplicated genes had relative close phylogenetic relationship (see below). Therefore, segmental and large-scale tandem duplication events appeared to have exclusively contributed to the expansion of the *OsPTR *gene family. Similar manner of member duplication had also been described in some large gene families in rice, for instance, the ankyrin repeat gene family [38] and the bHLH family [39].

### Phylogenetic analysis and multiple sequence alignment

**Figure 2:**
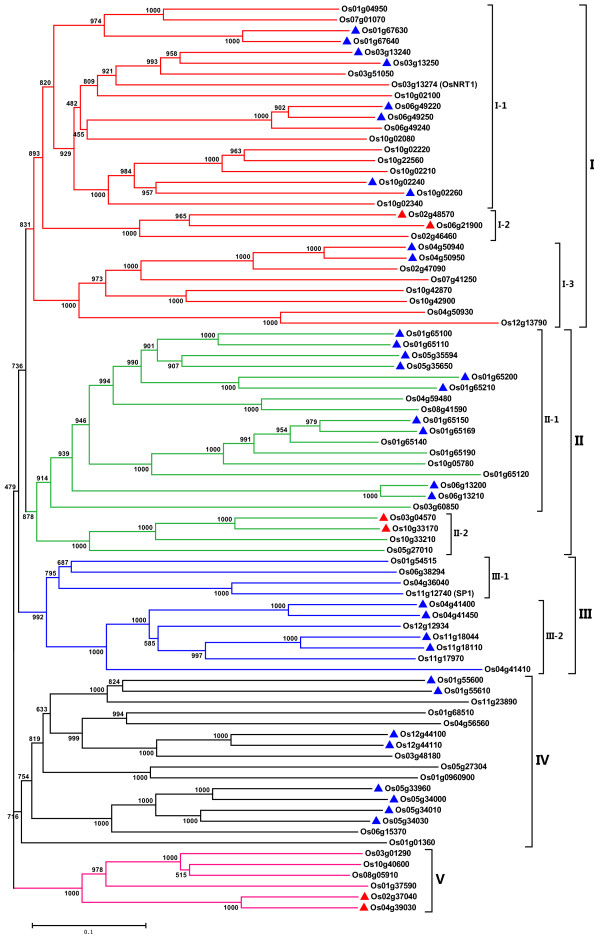
**Phylogenetic relationship and subfamily formation of OsPTR proteins**. Bootstrap values from 1000 replicates are indicated at each node. Scale bar represents 0.1 amino acid substitution per site. The proteins on the tree can be divided into five distinct subfamilies (I-V). Subfamily I, II and III are further divided into seven groups (I-1, I-2, I-3, II-1, II-2 and III-1, III-2). The paralogs whose corresponding genes involved in the duplication are marked with triangles (blue for tandem duplication and red for segmental duplication). The branches of different subfamilies are marked by different colors.

To examine in detail the phylogenetic relationship and functional divergence of OsPTR members, the aligned 84 OsPTR protein sequences were used to construct the joint unrooted phylogenetic tree. Our result suggested that the OsPTR proteins could be classified into five major subfamilies designated from I to V, with high bootstrap value support (Figure 2). This exercise resulted in five distinct subfamilies similar to that in *Arabidopsis *reported by Waterworth and Bray [2]. Within the five subfamilies formed, subfamily I, II and III were found to be more closely related to each other in comparison to subfamily IV and V. Subfamily I had the largest 30 PTR members and fell into three groups. Subfamily II, containing 21 PTR members was further divided into two groups. Subfamily III also including two groups contained 11 members. Subfamily IV had 16 members and subfamily V consisted of six members which had relative phylogenetic distance from other members. We identified 29 pairs of *OsPTR *genes that were close paralogs on the terminal node of phylogenetic tree. Tandem duplication contributed to 16 pairs of them and segmental duplication contributed to three pairs. These results furthermore illustrated that segmental and tandem duplication events were the dominant pattern in the expansion of *OsPTR *family members. To well understand the evolutionary history of *OsPTR *family, the amino acid sequences of PTR domains in each OsPTR protein were used for further phylogenetic analysis and similar tree was obtained. This result indicated that the main characteristic of *OsPTR *family was determined by the PTR domain.

In order to investigate the relationship between the function and phylogenetic subfamily formation of PTR members as well as identify some orthologous genes, a combined phylogenetic tree with OsPTR and AtPTR proteins was also established (Additional file 4). As a result, similar subfamilies were formed compared to the tree of OsPTR. Each subfamily contained both *Arabidopsis *and rice PTR members. However, most of the members were clustered in species-specific distinct clades, and only four pairs of orthologs (Os01g01360 and At5g13400, Os01g37590 and At2g26690, Os06g15370 and At1g68570, Os05g27010 and At2g40460) could be figured out. This result indicated that the main characteristics of PTR family in rice and *Arabidopsis *were formed before the split of monocotyledonous and dicotyledonous plants and then evolved separately in a species-specific manner. Moreover, the difference in the total number of OsPTR and AtPTR was mainly due to the variation in the number of subfamily I genes, which were 30 in rice and eight in *Arabidopsis*. Of the 30 subfamily I PTRs in rice, 21 were tandemly or segmentally duplicated genes.

**Figure 3:**
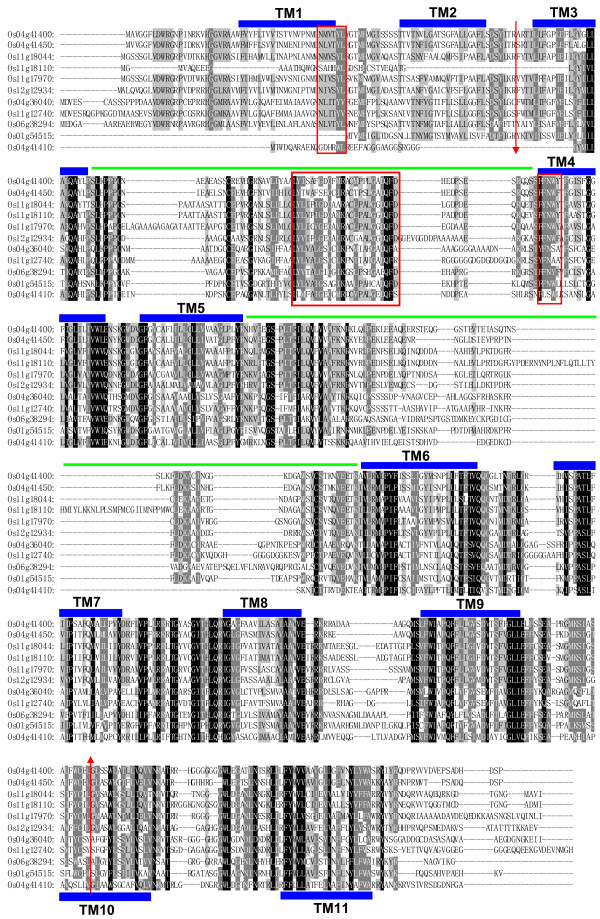
**Phylogenetic relationship and subfamily formation of OsPTR proteins**. Identical, conservative and block of similar amino acid residues are shaded in black, charcoal gray and gray, respectively. The TM regions are marked by blue rectangles. Green lines represent the hydrophilic loops in the PTR domain. The red arrows indicate the start and end position of PTR domain. The three conserved motifs are marked by red rectangles.

The alignments and comparison of the OsPTR full-length protein sequences and the PTR domain sequences illustrated that most of the amino acids in TM regions in or out of the PTR domain in members of the same phylogenetic subfamily were very conservative. On the contrary, the amino acids outside the TM regions were in great variation. In addition, some loop areas between the TM regions in the PTR domain of different members varied significantly both in length and amino acid composition. The alignment of the subfamily III members is shown in Figure 3 as an example. Due to the conservatism of the PTR domain and function variety of the PTR members, the diversity of the amino acids in the loop and beyond the PTR domain areas might be the major force of functional discrepancy of each PTR member.

**Figure 4:**
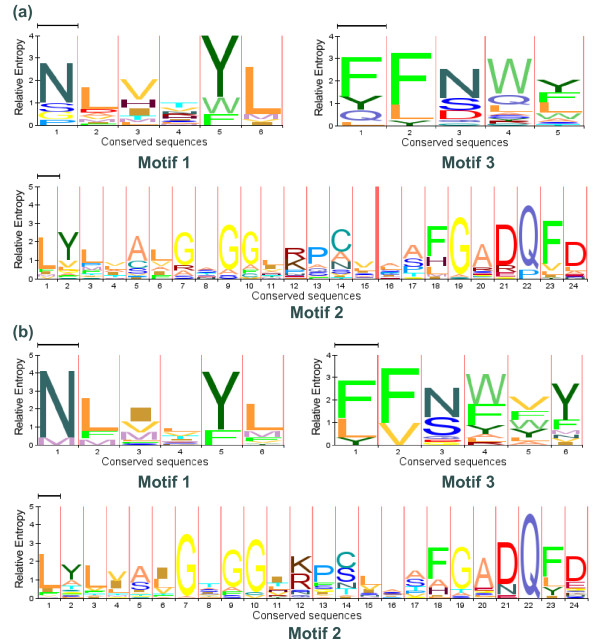
**HMM logos of conserved motifs in OsPTR and AtPTR**. X-axis indicates the conserved sequences of the motif. Y-axis is scales of relative entropy which reflect the conservative rate of each amino acid. The names of the motifs are marked under the graphics. (a) Logos of three motifs in OsPTR. (b) Logos of three motifs in AtPTR.

From the comparison of the protein sequences and sequences alignment, we found three special motifs that were highly conserved in most of the OsPTR members. Motif 1 (NLVxYL) was found nearly before the N terminal of the PTR domain. Motif 2 whose sequence was LYLxxxGxGGxK(R)xxxxxFGADQFD was located at the end of the first TM region of the PTR domain or stretched into the following hydrophilic loop before the second TM region. Following the motif 2, before/in or at the end of the second TM region of the PTR domain, motif 3 (FFNWY) was identified. The HMM logos of these motifs in OsPTR are shown in Figure 4a and their locations can be found in Figure 3. In most AtPTR members, similar conserved motifs could also be figured out (Figure 4b). However, motif 2 and 3 in AtPTR were located between the second and third TM regions of the PTR domain. Sequence screening of motif 2 in InterProscan and Uniprot databases retrieved only PTR family members. Therefore, we supposed that this motif was a newly identified signature sequences associated to the plant PTR family.

### Expression profiling of *OsPTR *genes in whole-life cycle of rice

**Figure 5:**
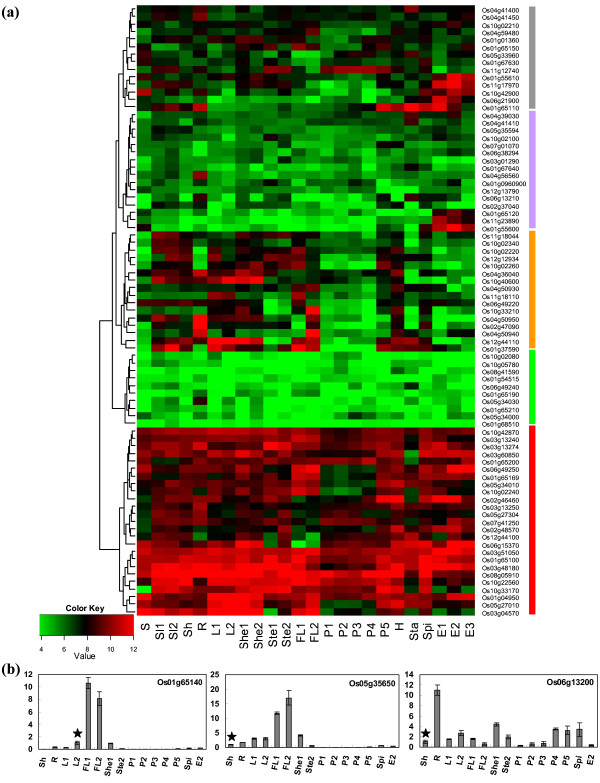
**Expression profile analysis of *OsPTR *genes**. (a) Hierarchical cluster display of expression profile for 81 *OsPTR *genes with corresponding probes in MH63. Color key represents log2 expression values. Samples are mentioned at the bottom: S, germinating seed at 72-h of imbibitions; Se1, Seedling of 3-day after sowing; Se2, Seedling of trefoil stage; Sh, R, shoot and root of seedling with two tillers, respectively; L1, leaf at secondary branch primordium differentiation stage; L2, leaf at meiosis stage; She1, sheath at secondary branch primordium differentiation stage; She2, sheath at meiosis stage; Ste1, stem at 5-day before heading; Ste2, stem at heading stage; FL1, flag leaf at 5-day before heading; FL2, flag leaf at 14-day after flowering; P1, P2, panicle at secondary branch and pistil/stamen primordium differentiation stage, respectively; P3, panicle at pollen-mother cell formation stage; P4, panicle at meiosis stage; P5, panicle at heading stage; H, hull of one day before flowering; Sta, stamen of one day before flowering; Spi, spikelet of 3-day after flowering; E1, E2, E3, endosperm of 7-day, 14-day and 21-day after pollination, respectively. On the left, cluster dendrogram is shown. On the right, five groups are marked with different rectangles. (b) Real-time PCR results of three *OsPTR *genes which have no probe in MH63. X-axis represents the stages mentioned above. Y-axis represents relative expression level compared with control. Reference samples (marked with asterisk) are used to illustrate the expression pattern more clearly.

Probes for 81 of 84 *OsPTR *genes could be found in our expression database. To analyze the expression profiles of the *OsPTR *genes, the signal values of the 81 *OsPTR *genes represented on the array were extracted. Average signal values for the 81 *OsPTR *genes from two or three biological replicates of each sample are given in Additional file 5. A hierarchical cluster displayed of logarithm of average signal values for the 81 *OsPTR *genes in selected tissues in Minghui 63 (MH63) is presented in Figure 5a. Three genes (Os01g65140, Os05g35650 and Os06g13200) had no corresponding probe set. Therefore, real-time PCR was used to study the expression pattern of these genes (Figure 5b).

Based on hierarchical clustering, expression pattern of *OsPTR *genes could be divided into five major groups. Group 1 consisted of 25 genes which showed high expression levels in all the tissues expect six genes that showed relatively low expression in certain tissues (Os03g60850 in Sta, Os03g04570 in Ste1, P1, Sta and E3, Os06g15370 in FL1, FL2 and Ste1, Os10g02240 in P1-P3, Os10g33170 in Se, Sta, E1 and E3 and Os12g44100 in P1-P3). Conversely, group 2 contained 10 genes that showed low expression level in the whole life cycle. Group 3 was comprised of 16 genes that showed relatively high expression level in both vegetative tissues and hull. All these genes had low expression level in early panicle and endosperm stages. Group 4 comprised of 16 genes with medium expression level in all the tissues except that Os04g56560 had high expression in root, Os01g65120 in E2, Os11g23890 in E1-E2 and Os01g55600 in E1-E3. The rest 14 genes belonged to group 5 although these genes had different expression patterns. For example, Os04g41400 had high expression signal in root and stamen, Os04g59480 showed high expression in FL2, Os11g12740 had high expression in panicle and stem. Interestingly, Os01g65110, Os01g55610, Os11g17970, Os10g42900 and Os06g21900 all showed relatively high expression in endosperms. The expression patterns of most *OsPTR *genes in Zhenshan 97 (ZS97) were similar to that in MH63 and the corresponding hierarchical cluster was shown in Additional file 6.

**Figure 6:**
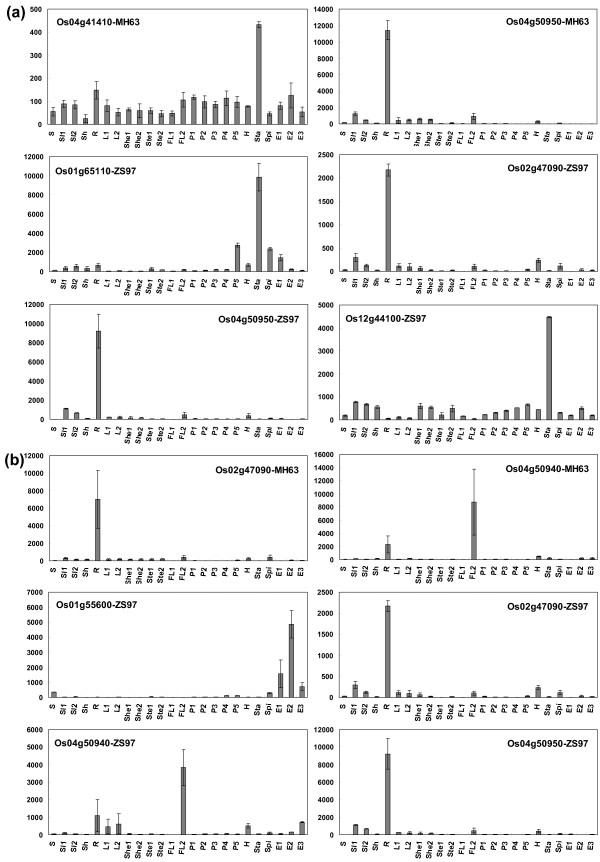
**Expression pattern of preferentially and tissue-specifically expressed *OsPTR *genes in MH63 and ZS97**. X-axis indicates representative samples and Y-axis is scales of expression level. Error bars showing the standard errors are based on biological replicates. (a) Preferentially expressed genes. (b) Tissue-specifically expressed genes. Detailed information of samples is listed in legend of Figure 5.

For further study regarding the expression pattern and looking inside the putative functions of *OsPTR *family members, we employed the analysis of preferential and tissue-specific expression genes in MH63 and ZS97. The identified preferential expression genes were mainly expressed in stamen and root. Os04g41410 was preferentially expressed in stamen of one day before flowering and Os04g50950 was in root of seedling with two tillers in MH63. In ZS97, Os01g65110 and Os12g44100 were identified as stamen preferential expression genes, while Os02g47090 and Os04g50950 were preferentially expressed in root (Figure 6a). The check of our whole genome expression profile of rice for different tissues obtained several tissue-specific expression genes including Os04g50940 in root of MH63 and in flag leaf of 14-day after flowering in MH63 as well as ZS97, Os02g47090 in root of MH63 and ZS97 and Os01g55600 in endosperm of 14-day after pollination in ZS97 (Figure 6b).

The hierarchical cluster of the *AtPTR *genes in 24 development stages released the expression profile of 51 genes except At1g72125, At3g45660 and At3g54450 (Additional file 7). Obviously, the expression patterns of *AtPTR *genes fell into the similar groups as *OsPTR *genes. Some tissue-specific expression *AtPTR *genes could also be identified, for example, At1g27040, At4g21680 and At3g25260 in seed and siliques, At3g45710 and At3g45700 in root. Most of the *AtPTR *genes had relatively low expression level in mature pollen except At1g62200, At5g28470 and At5g01180. These genes were most likely supplying nutrition to maturating pollen. Some of the orthologous *OsPTR *and *AtPTR *genes showed the similar expression pattern such as Os06g15370 and At1g68570, Os05g27010 and At2g40460. They both had high expression level in all the tissues. However, no more apparent relationship between the expression pattern and sequence similarities of OsPTR and AtPTR members could be pointed out.

### Expression regulation of *OsPTR *genes under phytohormone and light/dark treatments

**Figure 7:**
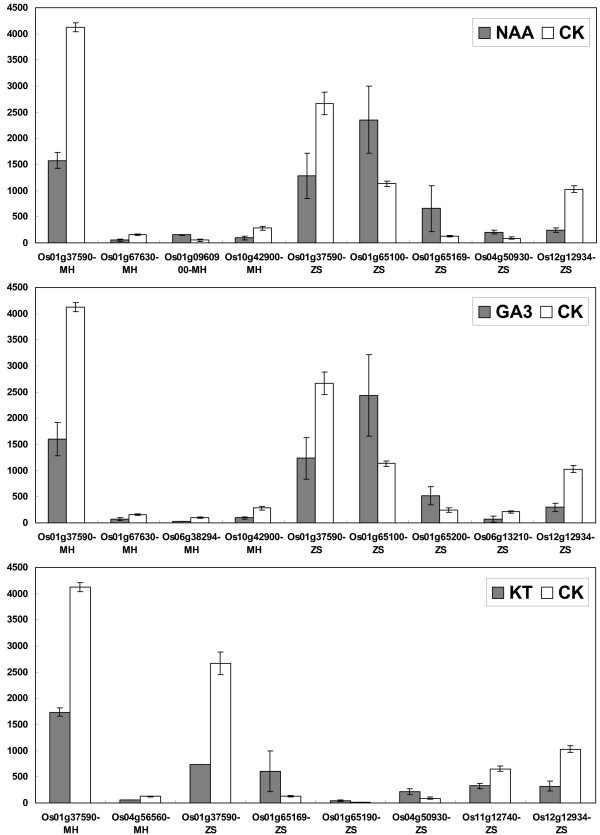
**Differential expressions shown by *OsPTR *genes in response to three phytohormone (NAA, GA3 and KT) treatments**. X-axis represents the differentially expressed genes. Y-axis represents average expression values obtained from microarrays. Each treatment is mentioned at the top of each diagram.

To determine the *OsPTR *genes in response to phytohormone treatments, microarray analysis was performed. We identified six and nine *OsPTR *genes that were differentially expressed under one or more of the phytohormone treatments compared with the control in MH63 and ZS97, respectively (Figure 7). Among these genes, only one gene (Os01g37590) was in common for two genotypes and it was down-regulated under three phytohormone treatments. The expression profile of the remaining genes in two genotypes was different. For instance, in MH63, Os01g67630 and Os10g42900 were down-regulated under NAA and GA3 treatments while Os04g56560 and Os06g38294 were down-regulated specifically to KT and GA3 treatment, respectively. Os01g0960900 was up-regulated specifically to NAA treatment. Five genes were up-regulated and four genes were down-regulated in ZS97. The up-regulated genes were Os01g65100, Os01g65169, Os01g65190, Os01g65200 and Os04g50930 while the down-regulated genes were Os01g37590, Os06g13210, Os11g12740 and Os12g12934. Os01g37590 and Os12g12934 were both down-regulated under three phytohormone treatments. Os01g65169 and Os04g50930 were both up-regulated under NAA and KT treatments. Os01g65100 was up-regulated under NAA and GA3 treatments. Os01g65190 and Os11g12740 showed differential expression specifically to KT treatment while Os01g65200 and Os06g13210 showed differential expression to GA3 treatment. The fold changes with respect to control of differential expression genes under phytohormone treatments are given in Additional file 8.

**Figure 8:**
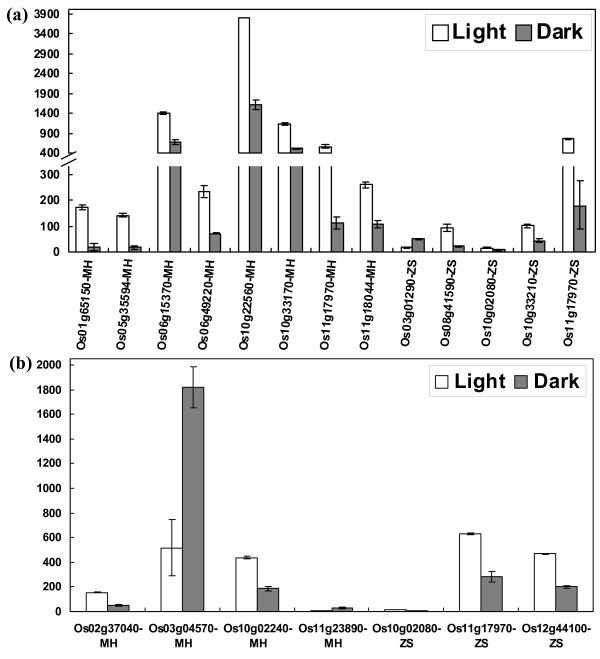
**Histogram diagrams of differentially expressed *OsPTR *genes regulated by light/dark treatments in MH63 and ZS97**. X-axis represents the differentially expressed genes. Y-axis represents average expression values gained from microarrays. Each treatment is mentioned at the top of each diagram. (a) Differential expression genes in plumule of 48 h after emergence with light/dark treatments. (b) Differential expression genes in radicle of 48 h after emergence with light/dark treatments.

To investigate possible light regulation of *OsPTR *genes, the expression profile of *OsPTR *genes in seedlings (plumule and radicle) treated with light or dark for 48 hours after emergence was also analyzed. Twelve and seven genes were differentially expressed under light compared to dark in plumule and radicle, respectively (Figure 8a and 8b). Eight genes (Os01g65150, Os05g35594, Os06g15370, Os06g49220, Os10g22560, Os10g33170, Os11g17970 and Os11g18044) showed differential expression in plumule and four genes (Os02g37040, Os03g04570, Os10g02240 and Os11g23890) in radicle in MH63. Interestingly, all the differentially expressed genes were up-regulated in plumule under light compared to dark while two of four genes in radicle were down-regulated. Os01g65150 was up-regulated nearly 10-fold under light compared to dark in plumule. In ZS97, five genes (Os03g01290, Os08g41590, Os10g02080, Os10g33210 and Os11g17970) were differentially expressed in plumule and three genes (Os10g02080, Os11g17970 and Os12g44100) in radicle. Interestingly, two genes (Os10g02080 and Os11g17970) were differentially expressed in both plumule and radicle in ZS97. The fold changes with respect to control of light/dark regulation genes are listed in Additional file 8.

### *Ka/Ks *analysis and express pattern divergence of paralogous *OsPTR *genes involved in duplication

To explore the mechanism of gene divergence after duplication, the coding sequences of the paralogous *OsPTR *genes involved in duplications were used to calculate the synonymous (*Ks*) and non-synonymous (*Ka*) substitution rate after duplication. The *Ka/Ks *ratio provides information about the degree of positive selection across all nucleotides in a gene since divergence. Generally, *Ka/Ks *= 1 means neutral selection, *Ka/Ks *< 1 indicates purifying selection and *Ka/Ks *> 1 shows the accelerated evolution with positive selection [40]. The *Ka/Ks *analysis demonstrated that *Ka/Ks *ratios for all the paralogous *OsPTR *genes were always less than one, most of them even close to zero, suggesting that purifying selection might be largely responsible for function maintenance of this family (Table 1).

**Table 1:** *Ka/Ks *and divergence analysis of paralogous *OsPTR *genes involved in duplication

Paralog pair	*Ka*	*Ks*	*Ka*/*Ks*	Purifying selection	Diver- gence model	Duplicate type	Sub- family	Protein identity (%)	Interval genes
Os02g37040	0.119	1.333	0.0892	yes	NF	Segmental	V	76.1	/
Os04g39030									
Os02g48570	0.247	10.084	0.0245	yes	NF	Segmental	I	55.0	/
Os06g21900									
Os03g04570	0.137	2.312	0.0593	yes	SF	Segmental	II	70.6	/
Os10g33170									
Os01g55600	0.412	57.191	0.0072	yes	D	Tandem	IV	44.0	0
Os01g55610									
Os01g65100	0.178	1.721	0.1035	yes	NF	Tandem	II	64.3	0
Os01g65110									
Os01g65150	0.090	0.370	0.2429	yes	D	Tandem	II	82.5	0
Os01g65169									
Os01g65200	0.304	1.141	0.2662	yes	NOF	Tandem	II	30.1	0
Os01g65210									
Os01g67630	0.087	0.525	0.1653	yes	NF	Tandem	I	61.6	0
Os01g67640									
Os03g13240	0.233	1.981	0.1175	yes	SF	Tandem	I	62.6	0
Os03g13250									
Os04g41400	0.153	0.975	0.1565	yes	SF	Tandem	III	75.2	3
Os04g41450									
Os04g50940	0.085	0.611	0.1397	yes	SF	Tandem	I	82.1	0
Os04g50950									
Os05g33960	0.183	1.262	0.1447	yes	NOF	Tandem	IV	62.6	3
Os05g34000									
Os05g34010	0.183	3.869	0.0473	yes	NOF	Tandem	IV	65.4	1
Os05g34030									
Os05g35594	0.247	1.884	0.1313	yes	/	Tandem	II	55.4	3
Os05g35650									
Os06g13200	0.038	0.274	0.1371	yes	/	Tandem	II	89.5	0
Os06g13210									
Os06g49220	0.082	0.387	0.2124	yes	NOF	Tandem	I	79.9	2
Os06g49250									
Os10g02240	0.290	1.856	0.1565	yes	NF	Tandem	I	56.5	1
Os10g02260									
Os11g18044	0.149	0.675	0.2201	yes	SF	Tandem	III	65.4	3
Os11g18110									
Os12g44100	0.177	0.592	0.2988	yes	NF	Tandem	IV	70.9	0
Os12g44110									

*Ka*: non-synonymous substitution rate. *Ks*: synonymous substitution rate. NF: neofunctionalization. SF: subfunctionalization. NOF: nonfunctionalization. D: just after duplication.

**Figure 9:**
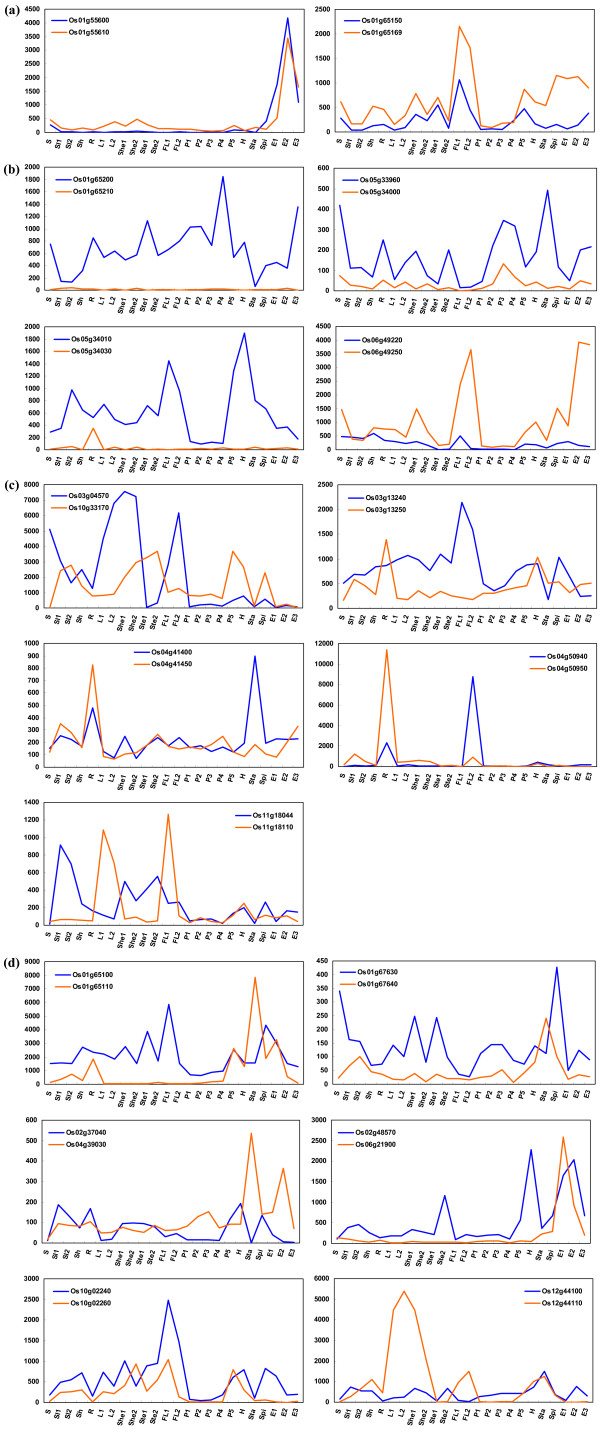
**Expression divergence of paralogous *OsPTR *genes involved in duplication**. X-axis indicates representative samples and Y-axis is scales of expression level. (a) Just formed due to duplication. (b) Nonfunctionalization. (c) Subfunctionalization. (d) Neofunctionalization.

Comparison of expression pattern shifts of the duplicated paralogous genes can reflect the divergence hypotheses that a duplicate gene pair might be involved in: nonfunctionalization, subfunctionalization and neofunctionalization [41]. The expression profile of the 17 pairs of paralogous *OsPTR *genes with corresponding probes was extracted for analysis. Obviously, the 17 pairs of paralogous *OsPTR *genes had divergent expression patterns. It seemed that all the three hypotheses influence the fate of the duplicated genes. Two pairs of genes (Os01g55600 and Os01g55610, Os01g65150 and Os01g65169) showed almost the same expression pattern in both copies, indicating they were just formed due to duplication (Figure 9a). The fate of four pairs in Figure 9b could be described as nonfunctionalization, where one copy of the paralog lost expression in all tissues. Subfunctionalization explained for five pairs of paralogous genes, in which the expression patterns of both copies were complementary to the pattern before duplication (Figure 9c). The divergence hypotheses of the rest six pairs of paralogous genes were assigned as neofunctionalization, where the expression of one copy had an obvious increase in one or more tissues (Figure 9d).

## Discussion

Di-/tripeptides and nitrate transport is important for various aspects of plant growth and development, in which peptide transporter family members may play important roles. Considering the potential functional diversity of PTR members associated with nutrient transport and only a few PTR family members have been described in higher plants, it was timely and quite relevant for us to characterize the rice PTR family. Combination of the whole genome sequence data, sequences alignment and high-throughput expression analysis undertaken in this investigation provides an insight into all the 84 members of PTR family in rice.

### Duplication played an important role in the expansion of the *OsPTR *family

It is well known that gene duplication is important for gene family evolution and diversity, which can occur via three major mechanisms: segmental duplication, tandem duplication and retroposition [42]. Due to repetitive plant genome duplications, certain plant genomes are likely to harbor relatively large members of a gene family, as compared to animal genomes [43]. The PTR families in rice and *Arabidopsis *are very much in line with the statement and showed increased family members in order to adapt for specific functions. The number of 84 OsPTR and 53 AtPTR members is far greater as compared to the low number of six in human, four in *C. elegans*, three in *Drosophila *and two in yeast [44]. It may indicate the importance of these transporters in plant growth and metabolism. Nearly 70% of the 84 *OsPTR *genes are duplicated genes, 21 genes (25%) are found to be involved in the segmental duplication while 44 (53.4%) participated in tandem duplication. Our analysis also indicated that in subfamily I, duplication events were largely responsible for the number difference of the member between OsPTR and AtPTR. It demonstrates that the *OsPTR *family may undergo extensive and rapid genomic changes which can maintain relative large number of member and lead to expression divergences between duplicate genes, further contributing to the establishment of novel gene functions [45].

### Phylogenetic relationship and functional diversity among the OsPTR members

The phylogenetic analysis reveals that the *OsPTR *family can be further divided into five subfamilies. The combined phylogenetic tree of OsPTR and AtPTR showed similar subfamily formation whereas most PTR members were clustered in species-specific clades and only four pairs of orthologs could be identified. It suggests that the members of the PTR family have diverged from each other primarily due to speciation and then undergone significant changes since the monocot/dicot split.

However, highly conserved motifs have been identified in most members of AtPTR and OsPTR. We further adopted the sequence alignment including the PTR members in plant, animal, human and prokaryotic organisms. It was surprising to find that the three motifs could be found in most eukaryotic PTR members but changed a lot or lost in the prokaryotic PTR members, especially motif 2 and motif 3. Moreover, some members such as Os01g65120, Os04g50930, Os12g13790 and At2g38100 which had far phylogenetic relationship from other PTR members, had great amino acid variation in motif 2 and motif 3. We inferred that motif 2 and motif 3 might be important characteristic logos of the eukaryotic PTR members and played considerable roles in the function formation of the PTR family. Further research may employ the point mutations introduced into these motifs, which should cause a loss of function of the corresponding protein. This experimentation could indicate the essentiality of these motifs for the formation of the transporter pore and the interaction with the substrates.

The sequence similarity among the OsPTR members seemed to show little relationship with their putative transporter type classification. In addition, we found that gene structures, intron phases of *OsPTR *genes and distribution of TM regions in the proteins had no relationship with their phylogenetic subfamily and transporter type classifications. As most OsPTR proteins had PTR as their only recognizable domain (See Additional file 2), we adopted the MEME method [46], in order to identify some new motifs or domains. However, the conserved motifs investigated by MEME were all TM regions (data not shown). We infer that the substrate selection of OsPTR proteins may be decided by their three dimensional structures. The determination of the di-/tripeptide, nitrate and other type transporters in this family by mutagenesis or crystal structure studies should solve the puzzle that di-/tripeptides, nitrate and other substrates of PTR proteins are very different in structure but they share the same transporter family - PTR [6].

### Expression pattern divergence of the *OsPTR *genes

Microarray provides a rich resource for investigating the evolution of gene expression. The temporal and spatial expression patterns of *OsPTR *genes are useful information for establishing their putative functions. Our microarray analysis indicated that the expression patterns of the 81 *OsPTR *genes could be classified into five groups. We also identified some preferential or tissue-specific expression *OsPTR *genes. Of the 81 genes analyzed in this study, only seven were found to exhibit either preferential or tissue-specific expression during different development stages of MH63 and ZS97. Interestingly, these genes were most preferentially or specifically expressed in root and stamen. Os02g47090, Os04g50940 and Os04g50950 were preferentially expressed in root. Moreover, these three genes were in the same phylogenetic clade that belonged to the group three of subfamily I. In *Arabidopsis*, the expression of *AtNRT1.1 *was found concentrated in the tips of primary and lateral roots [26]. *AtNRT1.2 *was primarily expressed in root hairs and the epidermis in both young and mature regions of roots [27]. *OsNRT1 *was constitutively expressed in the most external layer of the root, epidermis and root hair [34]. Our data showed that *OsNRT1 *had high expression levels in all the tissues especially in root. Os03g13240 and Os03g13250 were the paralogs of *OsNRT1*. Moreover, these three genes formed a tandem duplication cluster. Os03g13240 and Os03g13250 showed similar expression patterns with *OsNRT1 *and Os03g13250 had the highest expression level in root. It can be inferred that these root-preferentially or -specifically expressed *OsPTR *genes might be nitrate transporters and play important roles in root formation and development. *AtNRT1.4 *was expressed in the leaf petiole and participated in regulating leaf nitrate homeostasis [28]. The ortholog of *AtNRT1.4 *in rice was Os01g37590 which continuously expressed in vegetative tissues with high level in leaves. Expression of Os04g50940 was specific in flag leaf of 14-day after flowering both in MH63 and ZS97. Therefore, we infer that Os01g37590 and Os04g50940 might be participated in the development of leaf as well as the nutrient transporting in and/or out of leaf.

In addition, we also identified three genes (Os01g65110, Os04g41410 and Os12g44100) that were preferentially expressed in stamen and one gene (Os01g55600) in endosperm. One interesting phenomenon in our dendrogram was that three-quarter of the *OsPTR *genes had significant low expression level in panicles of different development stages. *SP1 *whose transcription accumulated in panicle was one of the exceptions. *SP1 *was highly expressed in the phloem of the branches of young panicles, which was consistent with the predicted function of *SP1 *and the phenotype of *sp1 *mutant [35]. It can be demonstrated that some PTR members might also play roles in nutrient transport in reproductive organ development.

### Potential involvement of OsPTR members in phytohormone and light/dark regulations

Little attention has been paid on the functions of PTR members in phytohormone and light/dark regulations. Until now, *AtNRT1.1 *was the only reported PTR gene that was involved in these regulations. *AtNRT1.1 *was regulated by auxin in both shoots and roots and involved in light-induced stomatal opening [47,48]. However, our microarray data showed that the expression patterns of more than one-third *OsPTR *genes changed significantly under phytohormone and light/dark treatments. Fourteen *OsPTR *genes are regulated by at least one phytohormone and 17 *OsPTR *genes are involved in the regulation of light/dark treatment in MH63 and ZS97. From these results, it can be speculated that these differentially expressed *OsPTR *genes may play an important role in the transport of regulatory molecules (e.g. hormone-peptide conjugates) and other metabolites induced by regulation of phytohormone and light/dark treatments. For example, IAA is an important plant growth phytohormone and 95% of the IAA pool is sequestered in an inactivated conjugated form, e.g. IAA can be conjugated to single amino acids, peptides or proteins via amide bonds. These conjugates may be involved in regulating phytohormone activity in response to plant growth and development, as well as facilitating hormone transport [49]. It seems that the proteins of those *OsPTR *genes regulated by NAA might have IAA conjugates transport activity.

### Fate of the paralogous *OsPTR *genes after duplication

From the *Ka/Ks *analysis of the paralogous *OsPTR *genes, it is obvious that purifying selection plays an important role in function maintenance of this family. However, comparison of the expression data implied that after duplication, the paralogous pairs may have been evolved in different hypotheses to gain functional divergence. Expression data shows that some of these duplicated genes have undergone subfunctionalization and neofunctionalization, which contribute to the possibility of maintenance and divergence of a paralogous set of genes. Some paralogs have adopted nonfunctionalization which contributes to the likelihood of loss of one member in a pair of paralogous genes. However, sufficient experimentation is required to prove these hypotheses.

## Conclusion

In conclusion, the results of this study display the genomic framework, characterization, conserved motifs, duplication manner and evolutionary analysis of the 84 OsPTR members, along with their expression profiles during whole-life stages as well as under phytohormone and light/dark treatments. These data will provide the basis for understanding the evolutionary history of PTR members and their roles in rice growth and development. The findings in our work would be useful in selecting candidate genes for functional validation studies of PTR members in rice. However, future research by adopting RNAi/overexpression strategies or insertion mutagenesis is required to elucidate the precise role of individual *OsPTR *gene.

## Methods

### Database screening and mining

Three methods were used to identify all putative PTR members in rice. First, domain search was executed on website of MSU RGAP http://rice.plantbiology.msu.edu/domain_search.shtml. Second, key words "PTR", "peptide transporter" and "proton-dependent oligopeptide transport" were used as queries to search against the annotation database of rice genome (Release 6.1 of MSU RGAP). Third, BLASTP and TBLASTN searches with the conserved sequence of PTR download from Pfam http://pfam.sanger.ac.uk/ as query were carried out in three databases: MSU RGAP http://rice.plantbiology.msu.edu/, National Center for Biotechnology Information (NCBI, http://www.ncbi.nlm.nih.gov/) and the Knowledge-Based Oryza Molecular Biological Encyclopedia (KOME, http://cdna01.dna.affrc.go.jp/cDNA). The parameters of blast searches were set as follows: max target sequences-500 and expect value less than 10. The Pfam search (E value < 1.0) and SMART http://smart.embl-heidelberg.de/ search (normal mode, including outlier homologues and homologues of known structure, Pfam domains, signal peptides, internal repeats and intrinsic protein disorder) were used to confirm the presence of PTR domain in each predicted protein. Information about the transcript, chromosomal position, full-length cDNA, BAC accession, gene structure for each gene and characteristics of proteins were obtained from MSU RGAP. The structure of each OsPTR protein was predicted in SMART. To predict the putative TM regions in each OsPTR protein, the TMHMM Server v2.0 http://www.cbs.dtu.dk/services/TMHMM/ was applied with default settings.

### Chromosomal localization and gene duplication

The physical positions of *OsPTR *genes were used to map these genes onto the corresponding rice chromosomes. The distribution of *OsPTR *genes on chromosomes was drawn by using the MapChart software [50] and modified manually with annotation. The presence of *OsPTR *genes on duplicated chromosomal segments was investigated by searching the segmental genome duplication of rice at MSU RGAP http://rice.plantbiology.msu.edu/segmental_dup/index.shtml with the maximum length distance permitted between collinear gene pairs of 500 kb. Gene distributed nearby and separated by five or fewer genes were considered to be tandem duplicates.

### Phylogenetic analysis and sequence alignment

ClustalX version 1.83 [51] was used to perform the multiple sequence alignment and generate the unrooted phylogenetic tree based on the protein sequences of OsPTR members with neighbor-joining method [52]. Bootstrap analysis was performed using 1000 replicates. The phylogenetic tree was displayed and annotated using MEGA software version 4 [53]. The combined tree with OsPTR and AtPTR proteins was generated using the same method. The multiple sequence alignment generated by ClustalX was modified manually and the conserved sequences were annotated according to the results of SMART and Pfam searches. The sequence alignment of the conserved motifs was submitted to the hmmbuild program of Mobyle [54] to build a profile HMM. Then the profile HMM was submitted into LogoMat-M [55] to draw the HMM logo.

### Rice materials and treatments

RNA samples used for microarray hybridization were obtained from two elite hybrid rice parents MH63 and ZS97 growing in normal field conditions at different developmental stages. For phytohormone treatments, seedlings at trefoil stage of MH63 and ZS97 were both treated with solutions of 0.1 mM NAA, GA3 and KT respectively. Samples were collected at the time points of 5, 15, 30 and 60 minutes after treatment and the samples under the same phytohormone treatment of different time points were mixed together. For light/dark treatments, seedlings at the plumule and radicle stages of MH63 and ZS97 were placed under 48 hours continuous light or darkness (when plumule was about 1-2 cm and radicle was about 2-3 cm in length) after emergence and harvested at the assigned time respectively.

### Microarray expression data analysis

Expression profile data of *OsPTR *genes was extracted from CREP database http://crep.ncpgr.cn of our lab that composed of hybridization of RNA samples from 39 tissues/organs covering the whole life cycle of rice with the Affymetrix rice microarray. The microarray data have been submitted under the accession number GSE19024 [56] into the NCBI Gene Expression Omnibus (GEO). Thirteen vegetative and 11 reproductive samples of different developmental stages that could cover the whole life cycle of rice were used for *OsPTR *expression profile analysis in this study. The detailed information of samples used in analysis is listed in Additional file 9.

Probe sets with the entire set of 11 probes (8-10 in some cases) present on the array with 100% identity over the corresponding genes were considered to be significant. Wherever more than one probe set was available for one gene, the probe set located on the 3' end was given preference. After normalization and variance stabilization, the average signal value of replicates for each probe set was used for analysis. Expression values of each gene were logarithmized in hierarchical cluster analysis and the heatmap were performed using R version 2.9.0 http://www.r-project.org/ with the method of complete linkage. The expression profile data of *AtPTR *genes was extracted from AtGenExpress (http://www.weigelworld.org/resources/microarray/AtGenExpress, [57]). Hierarchical cluster analysis and the heatmap drawing were used the same method as *OsPTR*.

To identify preferentially expressed genes, a student-*t *test was adopted. A gene was considered as preferential expression gene only if the signal value of the gene in a designated sample was more than two-fold and had a *P *value less than 0.05 as compared with the other samples. Also, a check of our whole genome expression profile data of rice for different tissues was used to identify tissue-specific expression genes [56]. Under phytohormone and light/dark treatments, genes that were up- or down-regulated more than two-fold with *P *value less than 0.05 as compared to control were considered as differential expression genes.

### Quantitative real-time PCR analysis

Real-time PCR reactions were carried out using the same RNA samples that were used for microarray. The sequences of primers used for real-time PCR are: Os01g65140 (F: AACTGGCTTCTGGCGAAAGAA, R: CGGAAGCAGCTTGACGAATAA), Os05g35650 (F: GGCAGCTGGTTCTCCAACAA, R: CGGCGATCAGCCAGTAGAAG), Os06g13200 (F: GCTCATGTTCGCGGTCATCT, R: TCCATCAACATCCCCTGCTT) and *Actin1 *(F: TGGCATCTCTCAGCACATTCC, R: TGCACAATGGATGGGTCAGA). First-stand cDNA was synthesized from DNaseI-treated total RNA using Superscript III reverse transcriptase (Invitrogen). Real-time PCR were performed in 25 μl reaction mixture with 1 μl of first-strand cDNA, 12.5 μl 2× SYBR Premix^® ^Ex Taq™ (TaKaRa), 0.5 μl 50× ROX reference dye II and 0.5 μl of each primer. The reactions were carried out using ABI PRISM 7500 system (Applied Biosystems). The following program was used: 95°C for 10 s; 45 cycles of 95°C for 5 s, 60°C for 34 s. Rice *Actin1 *was used as internal control. The relative expression levels were analyzed as described previously [58].

### *Ka *and *Ks *calculations

The paralogous PTR genes in rice were inferred from the phylogenetic tree analysis. Pairwise aligned paralogous protein sequences generated by the ClustalW2 http://www.ebi.ac.uk/Tools/clustalw2/ were used as the alignment guides. PAL2NAL (http://www.bork.embl.de/pal2nal/, [59]) was used to convert the multiple sequence alignment of proteins and the corresponding mRNA sequences into a codon alignment. Then the calculation of non-synonymous (*Ka*) and synonymous (*Ks*) substitution rates were carried out on the PAL2NAL website automatically by using the codeml program in PAML [60]. By performing the EMBOSS Pairwise Alignment Algorithms on EBI http://www.ebi.ac.uk/Tools/emboss/align/, we analyzed the protein identities between paralogs of OsPTR.

## Authors' contributions

XBZ designed and carried out most of the computational work, analyzed the data, performed the real-time PCR and drafted the manuscript. JYH made great efforts in data collection, analysis and helped to draft the manuscript. HHY participated in hierarchical cluster construction and preferential expression analysis. LW carried out the microarray experiment and WBX helped to analyze the expression data. All authors have read and approved the final manuscript.

## Supplementary Material

Additional file 1 The detailed information of each *OsPTR *gene and corresponding protein. Click here for file

Additional file 2 **Domain composition and structures of OsPTR proteins predicted by SMART**. The graphical presentations are: transmembrane segments as predicted by the TMHMM program (blue vertical rectangle); segments of low compositional complexity determined by the SEG program (pink rectangle); signal peptides determined by the SignalP program (red rectangle); disordered regions detected by DisEMBL (blue horizontal rectangle). The scale is in an amino acid. Click here for file

Additional file 3 **TM prediction result of OsPTR proteins by TMHMM**. The predicted TM regions are marked with red rectangle and their probabilities are list in the Y-axis. Length: the length of the protein sequence. Number of predicted TMs: The number of predicted TM helices. Total prob of N-in: The total probability that the N-term is on the cytoplasmic side of the membrane. Click here for file

Additional file 4 **Phylogenetic relationship of rice and *Arabidopsis *PTR proteins**. The unrooted tree was generated using ClustalX program by neighbor-joining method. Bootstrap values from 1000 replicates are indicated at each node. The orthologous proteins are marked with red circles and the AtPTR proteins are marked with blue triangles. The tree can be divided into five subfamilies: I to V. Click here for file

Additional file 5 Average microarray signal values of 81 *OsPTR *genes in MH63 and ZS97 Click here for file

Additional file 6 **Hierarchical cluster display of expression profile for 81 *OsPTR *genes with corresponding probes in ZS97**. Color key represents log2 expression values. Developmental stages (Additional file 9) used for expression profiling are mentioned on bottom of each column. On the left side of expression map, cluster dendrogram is shown. On the right side, five groups have been marked with different rectangles for the genes showing different expression patterns. Click here for file

Additional file 7 **Hierarchical cluster display of expression profile for 50 *AtPTR *genes with corresponding probes**. Color key at the left represents log2 expression values. Developmental stages (list in the table below) used for expression profiling are mentioned on bottom of each column. On the left side of expression map, cluster dendrogram is shown. Click here for file

Additional file 8 Results of differential expression analysis in 7 d-old seedlings subjected to three phytohormone (NAA, GA3 and KT) treatments and plumule or radicle with light/dark regulation in MH63 and ZS97 Click here for file

Additional file 9 Detailed information of rice samples used in microarray analysis Click here for file
