# Supplementary material for: Genomic survey, characterization and expression profile analysis of the peptide transporter family in rice (Oryza sativa L.)
Source: BMC Plant Biol. 2010 May 20;10:92. doi: 10.1186/1471-2229-10-92 (PMC3017762; doi:10.1186/1471-2229-10-92)
Supplement: Additional file 1 — The detailed information of each OsPTR gene and corresponding protein. [file 1471-2229-10-92-S1.PDF]

Additional file 1 − The detailed information of each *OsPTR* gene and corresponding protein

Member Name	KOME/NCBI Fl-cDNA	IN[b]	Chr[c]	RGAP V6.1 Genomic Position 5'-3'	Gene orient-ation[d]	BAC/ PAC	ORF Length (bp)	Protein			Gene structure [g]
								L[e] (aa)	MW[f] (Da)	pI	
Os01g01360	AK070557, AK058723	0	1	177310-179812	Down	AP002818	1821	606	66437.2	8.32	
Os01g04950	AK105664, AK070036, AK102216	3	1	2293510-2297407	Down	AP002483	1743	580	63097.4	6.66	
Os01g37590	/	1	1	21037774-21040035	Down	AP003372	1758	585	62577.7	8.33	
Os01g54515	AK242252	4	1	31355138-31357904	Down	AP003076	1587	528	58152.2	9.36	
Os01g55600	AK109619, AK105550	3	1	32033967-32031642	Up	AP003274	1725	574	62199.1	8.99	
Os01g55610	AK069522	2	1	32039334-32036974	Up	AP003274	1773	590	64559.7	9.13	
Os01g65100	AK068840	3	1	37789297-37791955	Down	AP003235	1638	545	58991.2	8.3	
Os01g65110	AK103248	3	1	37794553-37796703	Down	AP003235	1647	548	59124.5	9.42	
Os01g65120	/	3	1	37799913-37801760	Down	AP003235	1479	492	52539.8	11.4	
Os01g65140	/	1	1	37809727-37811086	Down	AP003235	1026	341	36469	7.92	
Os01g65150	AK102239	3	1	37813630-37815732	Down	AP003235	1614	537	56948.6	8.86	
Os01g65169	AK099405	3	1	37820175-37825971	Down	AP003235	1626	541	57498.2	8.14	
Os01g65190	AK243378	4	1	37827231-37831338	Down	AP004669	1989	662	70328	9.43	
Os01g65200	AK068086, AK066692, AK099762	4	1	37833134-37835883	Down	AP004669	1647	548	60003.3	7.24	
Os01g65210	AK060248	7	1	37836094-37842621	Down	AP004669	2139	712	77977.2	7.54	
Os01g67630	AK242900	3	1	39306400-39309538	Down	AP006757	1674	557	60403.3	7.99	
Os01g67640	/	5	1	39311687-39314648	Down	AP006757	1800	599	65697.9	9.74	
Os01g68510	AK100698, AK064467	3	1	39809260-39814163	Down	AP003436	1803	600	65788.9	7.96	
Os01g0960900[a]	NM_001052004[a]	3	1	42362118-42356430 [a]	Up	AP008207	1659	552	57608.6	9.22	
Os02g37040	AK120486, AK072036	4	2	22368547-22364038	Up	AP006161	1830	609	64675.9	8.88	
Os02g46460	AK065840	4	2	28310329-28306720	Up	AP004096	1833	610	66036.9	6.51	
Os02g47090	AK109931	2	2	28743728-28747228	Down	AP004778	1725	574	61286.8	5.01	
Os02g48570	AK287911	6	2	29735844-29730294	Up	AP004047	1755	584	64106	6.7	
Os03g01290	AK241823	2	3	201527-204750	Down	AC125411	1770	589	63965.2	8.95	
Os03g04570	AK072119	2	3	2135721-2130968	Up	AC118132	1803	600	65950.6	9.35	
Os03g13240	AK318551, CT835206	4	3	7142556-7138070	Up	AC137696	1776	591	65196.1	5.7	
Os03g13250	AK100552, CT831171	4	3	7150338-7146276	Up	AC137696	1866	621	68751.2	6.77	
Os03g13274	AK066920, AF140606, OsNTR1	5	3	7167863-7162369	Up	AC137696	1755	584	64797.1	6.72	

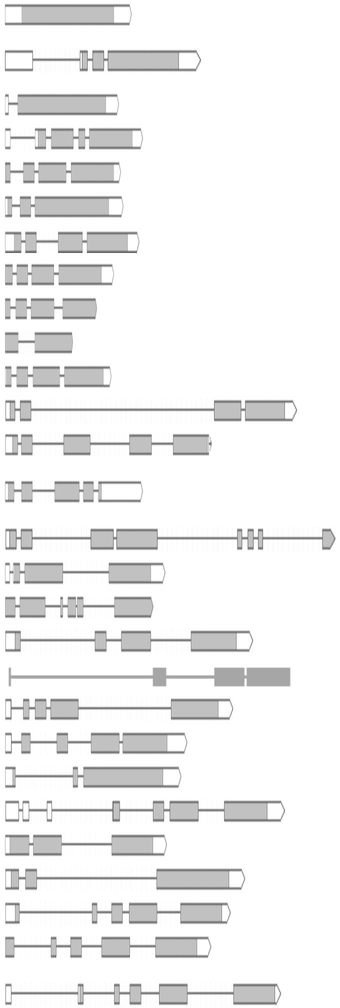

Os03g48180	AK065253, AK099321, AK104378, AK099244, AK099218, AK099910	2	3	27411830-27406523	Up	AC092779	1764	587	63562.8	9.1
Os03g51050	AK059693, AK070804, AK099012, AK072691, AB067652, CT829552	5	3	29183413-29178058	Up	AC147426	1782	593	65613.5	4.88
Os03g60850	AK058972, AK099193	3	3	34569843-34567177	Up	AC093018	1758	585	63730	8.78
Os04g36040	AK072763, AK064785	4	4	21803970-21798837	Up	BX548156	1836	611	64514.4	8.1
Os04g39030	AK111143	4	4	22997712-22993517	Up	AL663017	1779	592	62373.1	9.06
Os04g41400	/	4	4	24364033-24361202	Up	AL606613, AL731617	1719	572	62261.7	8.44
Os04g41410	/	3	4	24368261-24365407	Up	AL731617	1455	484	53205.1	7.21
Os04g41450	AK101116, AK068089	5	4	24381318-24375848	Up	AL731617	1674	557	62103.1	7.4
Os04g50930	/	5	4	29960062-29963226	Down	AL607004	1854	617	66860.7	5.65
Os04g50940	AK070216, AK105711	1	4	29971122-29974180	Down	AL607004	1728	575	62384.2	4.74
Os04g50950	AK101480, AY305030	3	4	29976322-29980387	Down	AL607004	1782	593	65165.8	5.32
Os04g56560	AK108874	2	4	33544692-33547387	Down	AL606608	1896	631	67824.3	9.48
Os04g59480	/	2	4	35174938-35178402	Down	AL606656	1533	510	54932.4	7.81
Os05g27010	AK108393	0	5	15653274-15651579	Up	AC144739	1512	503	54439.9	9.45
Os05g27304	CT836455	2	5	15808780-15816130	Down	AC134342	1752	583	61727.2	8.76
Os05g33960	AK110441	2	5	19988278-19982670	Up	AC130729	1815	604	65201.2	8.52
Os05g34000	AK108312	2	5	20022543-20025417	Down	AC130729	1923	640	68733.5	8.63
Os05g34010	AK070558, AK119621	2	5	20029009-20024154	Up	AC130729	1797	598	65489.1	9.81
Os05g34030	/	2	5	20036625-20038643	Down	AC130729	1857	618	66825.5	6.25
Os05g35594	AK068274, CT828758	2	5	21085804-21081775	Up	AC130600	1593	530	56615	8.42
Os05g35650	AK242663	3	5	21129181-21122038	Up	AC108502	1746	581	61771.2	8.43
Os06g13200	/	3	6	7234609-7230573	Up	AP004995	1566	521	56631.1	8.04
Os06g13210	AK064959	3	6	7246730-7250059	Down	AP004995	1608	535	57734.3	7.98
Os06g15370	AK120596	3	6	8738432-8743023	Down	AP004993	1794	597	65091	7.83
Os06g21900	AK242131	4	6	12654818-12658095	Down	AP004750	1929	642	70101.2	8.06
Os06g38294	AK242774, AK071803	3	6	22664092-22654618	Up	AP003630	1773	590	63732.1	8.99
Os06g49220	AK119527	3	6	29823345-29825788	Down	AP003761	1710	569	61717.8	6.73
Os06g49240	AK070685	1	6	29828477-29830126	Down	AP003761	1332	443	48072.2	7.91
Os06g49250	AK064899	4	6	29837285-29840402	Down	AP003761	1644	547	58588.5	5.43
Os07g01070	AK100112, AK106932	0	7	41657-43577	Down	AP005869	1719	572	61799.4	8.69
Os07g41250	AK101099, AK061822	4	7	24719073-24722879	Down	AP005809	2037	678	74415.4	7.71

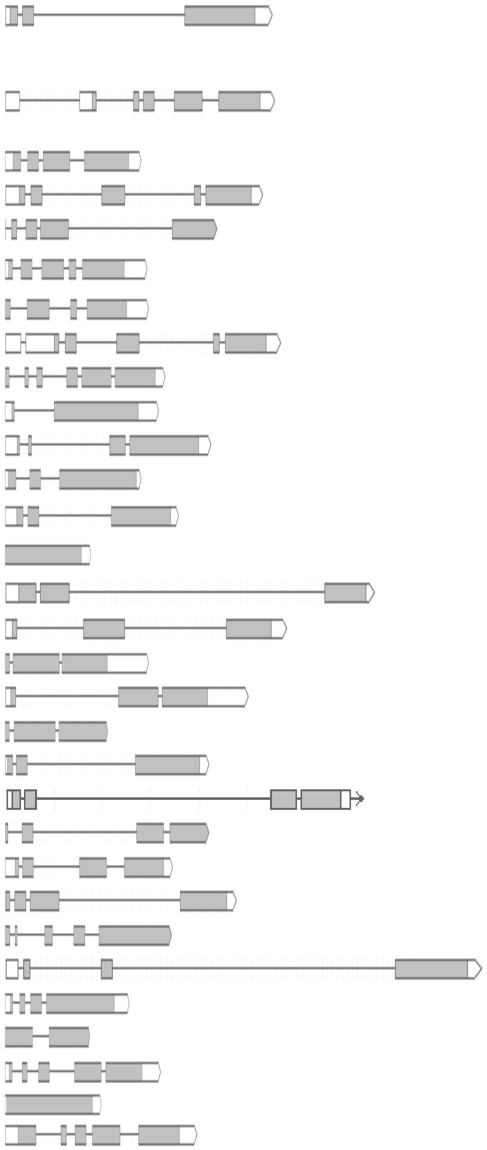

Os08g05910	AK061227, AK070077, AK120196, CT832571	2	8	3189454-3181312	Up	AP003905	1812	603	64837.3	9.23
Os08g41590	/	0	8	26268027-26266745	Up	AP003857	1167	388	41086.3	8.81
Os10g02080	/	2	10	682303-679075	Up	AC068654	1551	516	55555.4	8.46
Os10g02100	AK288596	4	10	694775-688266	Up	AC068654	1725	574	61694.4	7.37
Os10g02210	AK288351	7	10	764693-760157	Up	AC079685	1776	591	64989.6	7.27
Os10g02220	AK070621, AK071280, CT832467	5	10	775317-770655	Up	AC079685	1701	566	62274.1	6.7
Os10g02240	AK068679, AK066625, CT829352	4	10	790215-786877	Up	AC079685	1743	580	63035.8	8.58
Os10g02260	AK069771, AK065457	4	10	808777-804639	Up	AC079685	1731	576	63309.3	6.37
Os10g02340	/	1	10	835729-837970	Down	AC079685	1671	556	58920	5.49
Os10g05780	/	1	10	2913690-2911731	Up	AC091665	1662	553	58376.8	7.49
Os10g22560	AK065895	4	10	11623854-11619957	Up	AC107314	1713	570	62031.9	8.24
Os10g33170	AK105737, AK059075, AK101007	2	10	17303217-17307814	Down	AC026815	1824	607	65370	9.13
Os10g33210	AK101055	2	10	17344375-17349730	Down	AC026815	1833	610	66381.9	9.4
Os10g40600	AK105765, AK068409	1	10	21690678-21686244	Up	AC037426	1791	596	63723.9	8.14
Os10g42870	AK099219, AK070527, AK105726, AK099267	4	10	23043170-23046226	Down	AC018727	1710	569	61569	6.71
Os10g42900	AK121847, AK066937	1	10	23057470-23060637	Down	AC018727	1716	571	62294.3	9.23
Os11g12740	AK100802, SP1	4	11	7194297-7188975	Up	AC135258	1881	626	66064	6.88
Os11g17970	AK242720, AK111488	4	11	10090683-10097378	Down	AC133006	1812	603	64470.8	8.3
Os11g18044	AK105635, AK101066	4	11	10127922-10138581	Down	AC133006	1752	583	63250.5	8.36
Os11g18110	/	7	11	10179819-10186387	Down	AC133002	1710	569	61845.7	6.96
Os11g23890	/	5	11	13074991-13068725	Up	AC146913, AC145327	1959	652	72066.5	9.83
Os12g12934	AK120737	4	12	7163195-7168416	Down	AL954160	1752	583	62757.1	7.92
Os12g13790	/	5	12	7792875-7796966	Down	AL731786	1401	466	49147.2	6.11
Os12g44100	AK060510, AK068351	1	12	27317096-27312522	Up	AL731762	1767	588	62797.6	8.99
Os12g44110	AK068058	0	12	27327854-27325824	Up	AL731762	1764	587	64143.4	9.24

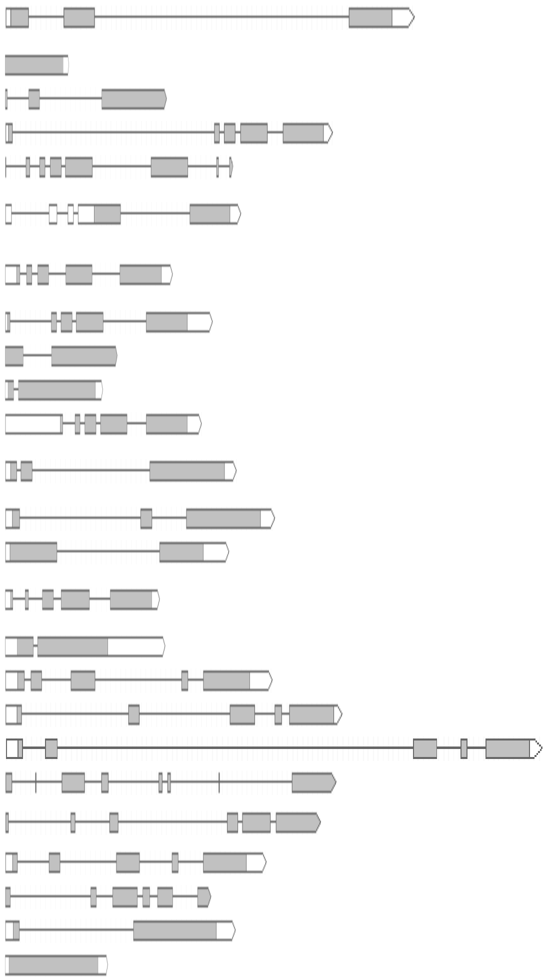

a: annotation of NCBI. b, IN: intron numbers. c, Chr: chromosome. d, Down: downward, Up: upward. e, L: length. f, MW: molecular weight. g: the coding and untranslated regions are represented by grey and white boxes, respectively. Lines represent the introns.
