# Supplementary material for: Genomic survey, characterization and expression profile analysis of the peptide transporter family in rice (Oryza sativa L.)
Source: BMC Plant Biol. 2010 May 20;10:92. doi: 10.1186/1471-2229-10-92 (PMC3017762; doi:10.1186/1471-2229-10-92)
Supplement: Additional file 2 — Domain composition and structures of OsPTR proteins predicted by SMART. The graphical presentations are: transmembrane segments as predicted by the TMHMM program (blue vertical rectangle); segments of low compositional complexity determined by the SEG program (pink rectangle); signal peptides determined by the SignalP program (red rectangle); disordered regions detected by DisEMBL (blue horizontal rectangle). The scale is in an amino acid. [file 1471-2229-10-92-S2.PDF]

Additional file 2 – Domain composition and structures of OsPTR proteins predicted by SMART

The graphical presentations are: transmembrane segments as predicted by the TMHMM program (blue vertical rectangle); segments of low compositional complexity determined by the SEG program (pink rectangle); signal peptides determined by the SignalP program (red rectangle); disordered regions detected by DisEMBL (blue horizontal rectangle).The scale is in an amino acid.

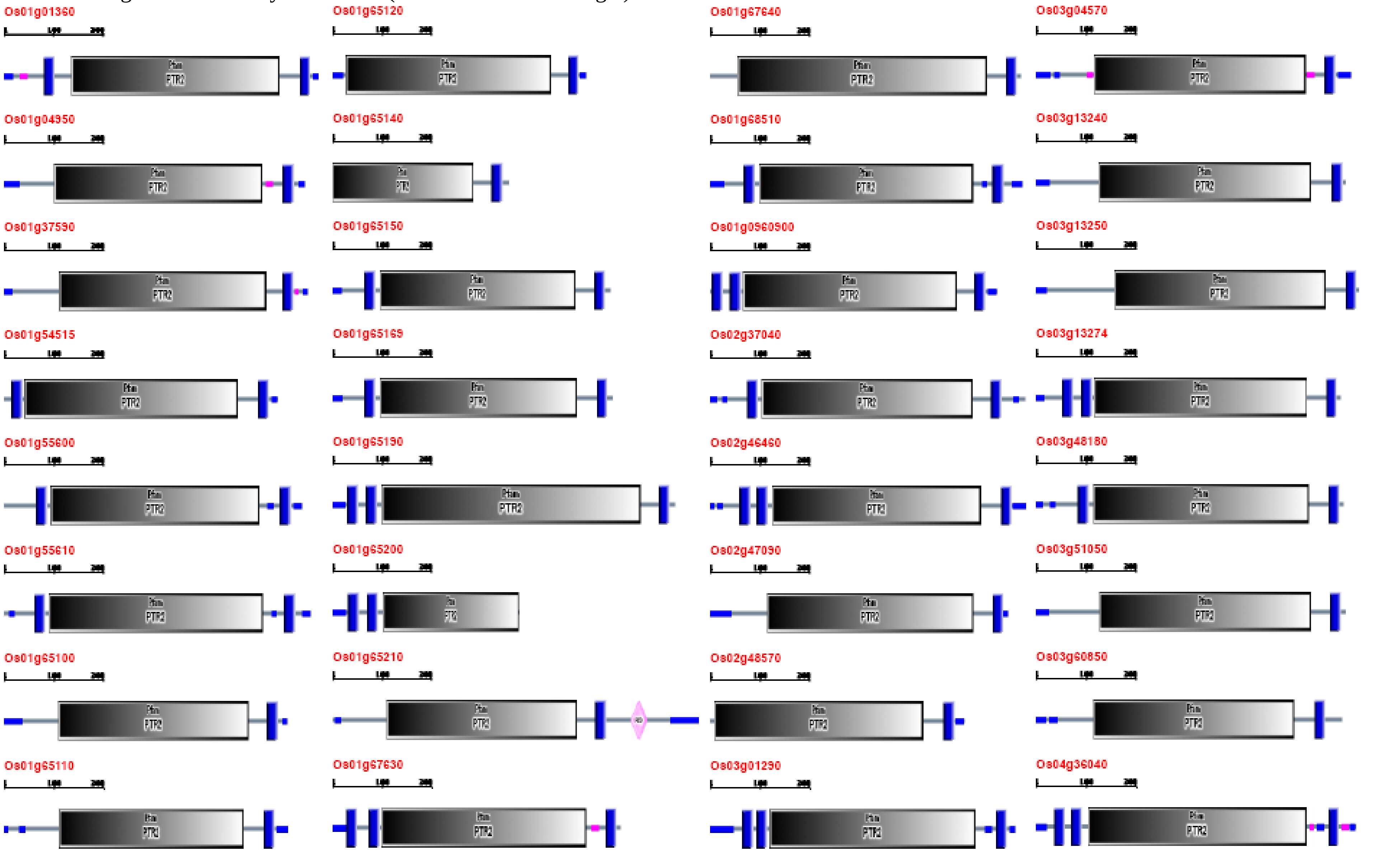

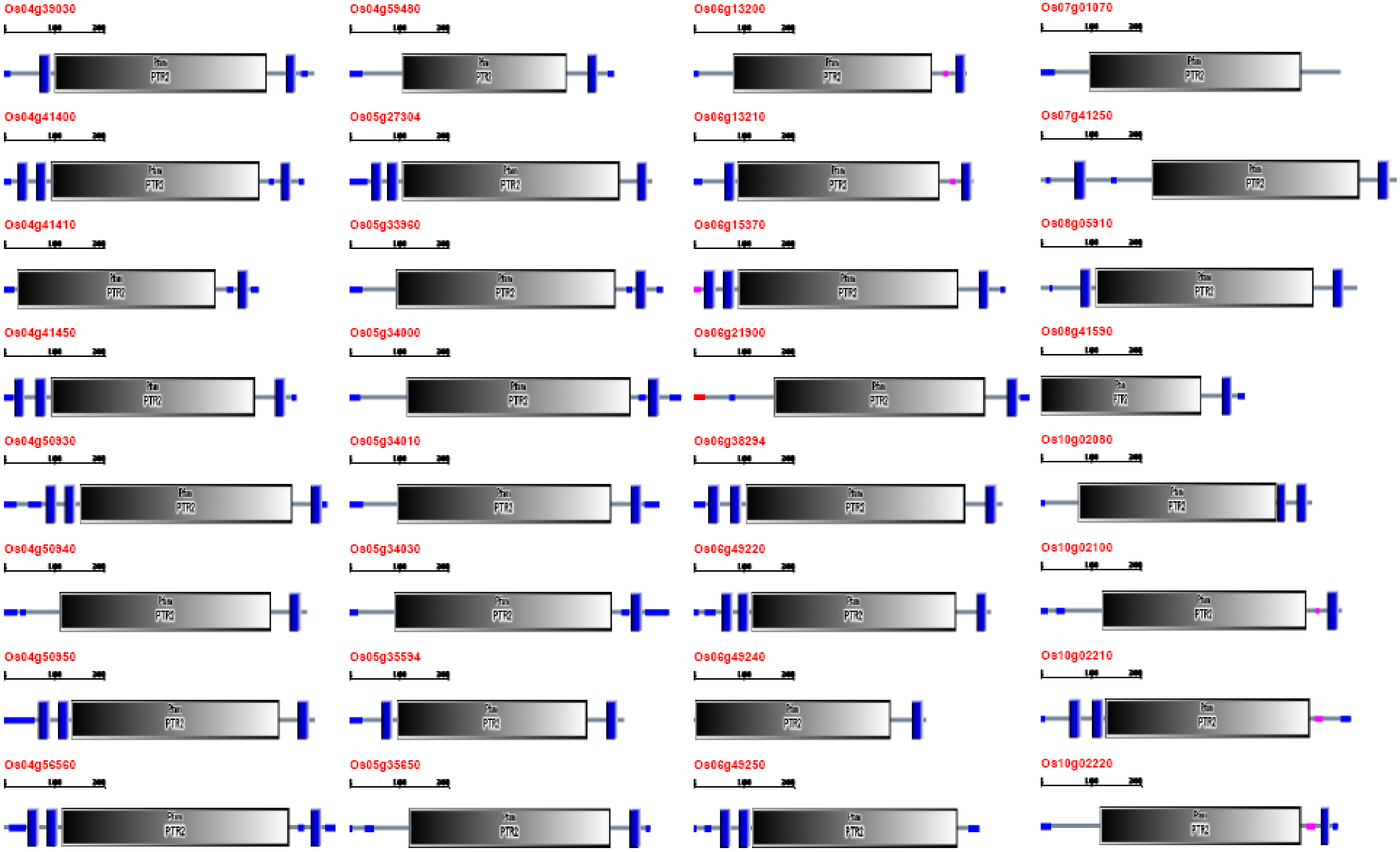
Os04g39030
Os04g41400
Os04g41410
Os04g41450
Os04g50930
Os04g50940
Os04g50950
Os04g56560
Os04g59480
Os05g27304
Os05g33960
Os05g34000
Os05g34010
Os05g34030
Os05g35594
Os05g35650
Os06g13200
Os06g13210
Os06g15370
Os06g21900
Os06g38294
Os06g49220
Os06g49240
Os06g49250
Os07g01070
Os07g41250
Os08g05910
Os08g41590
Os10g02080
Os10g02100
Os10g02210
Os10g02220

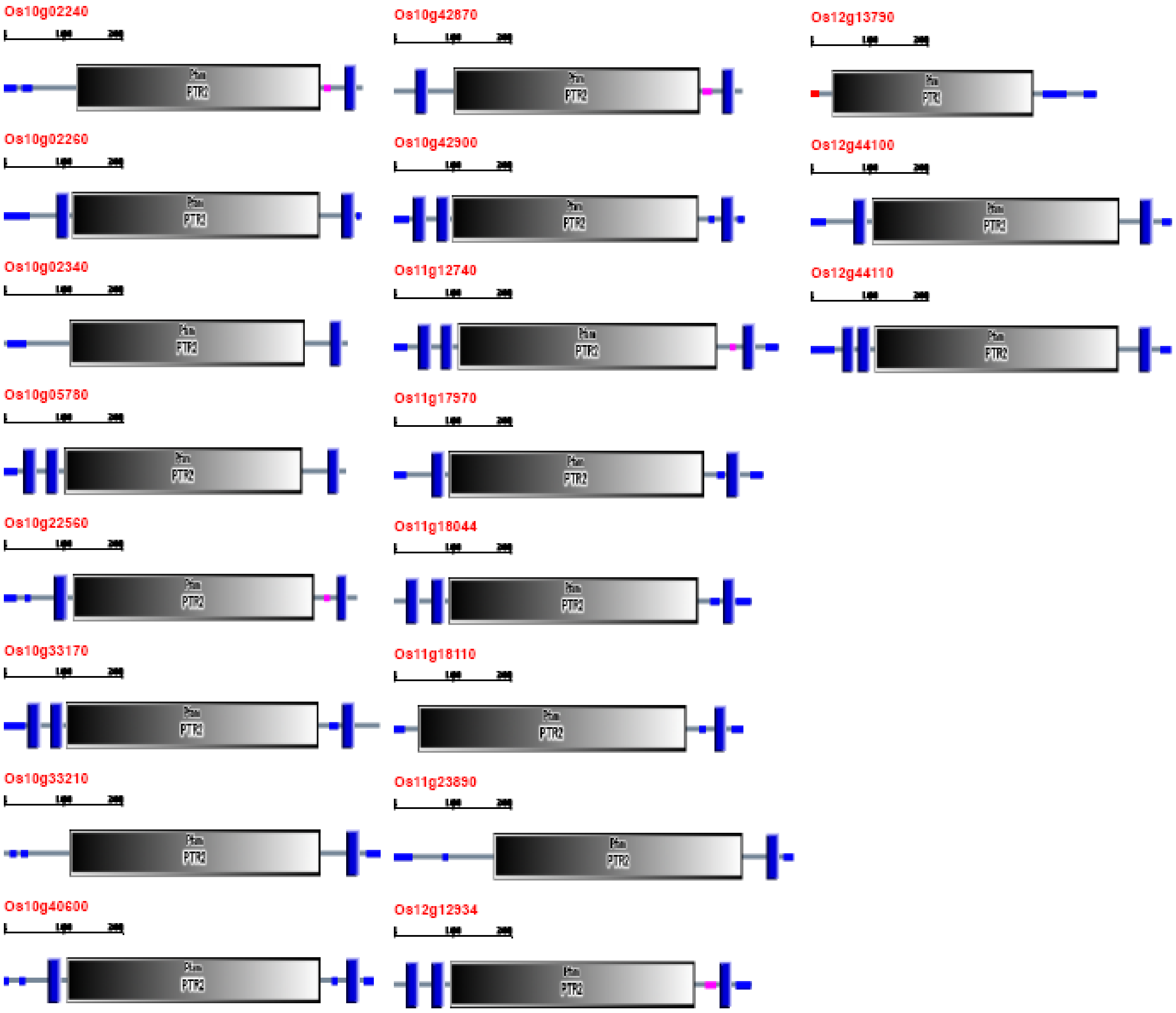
Os10g02240
Os10g02260
Os10g02340
Os10g05780
Os10g22560
Os10g33170
Os10g33210
Os10g40600
Os10g42870
Os10g42900
Os11g12740
Os11g17970
Os11g18044
Os11g18110
Os11g23890
Os12g12934
Os12g13790
Os12g44100
Os12g44110
