# Supplementary material for: Genomic survey, characterization and expression profile analysis of the peptide transporter family in rice (Oryza sativa L.)
Source: BMC Plant Biol. 2010 May 20;10:92. doi: 10.1186/1471-2229-10-92 (PMC3017762; doi:10.1186/1471-2229-10-92)
Supplement: Additional file 3 — TM prediction result of OsPTR proteins by TMHMM. The predicted TM regions are marked with red rectangle and their probabilities are list in the Y-axis. Length: the length of the protein sequence. Number of predicted TMs: The number of predicted TM helices. Total prob of N-in: The total probability that the N-term is on the cytoplasmic side of the membrane. [file 1471-2229-10-92-S3.PDF]

Additional file 3 – TM prediction result of OsPTR proteins by TMHMM

The predicted TM regions are marked with red rectangle and their probabilities are list in the Y-axis. Length: the length of the protein sequence. Number of predicted TMs: The number of predicted TM helices. Total prob of N-in: The total probability that the N-term is on the cytoplasmic side of the membrane.

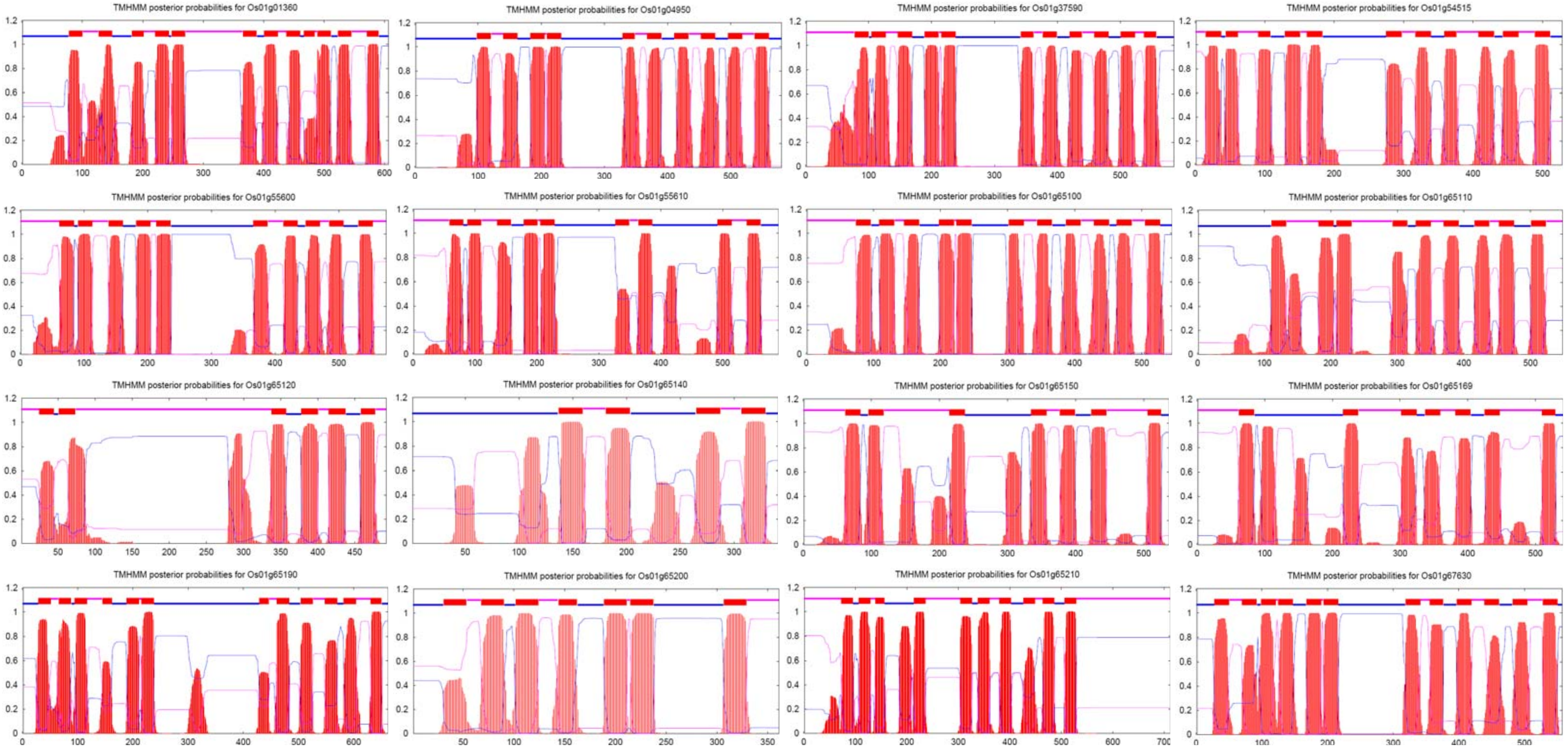

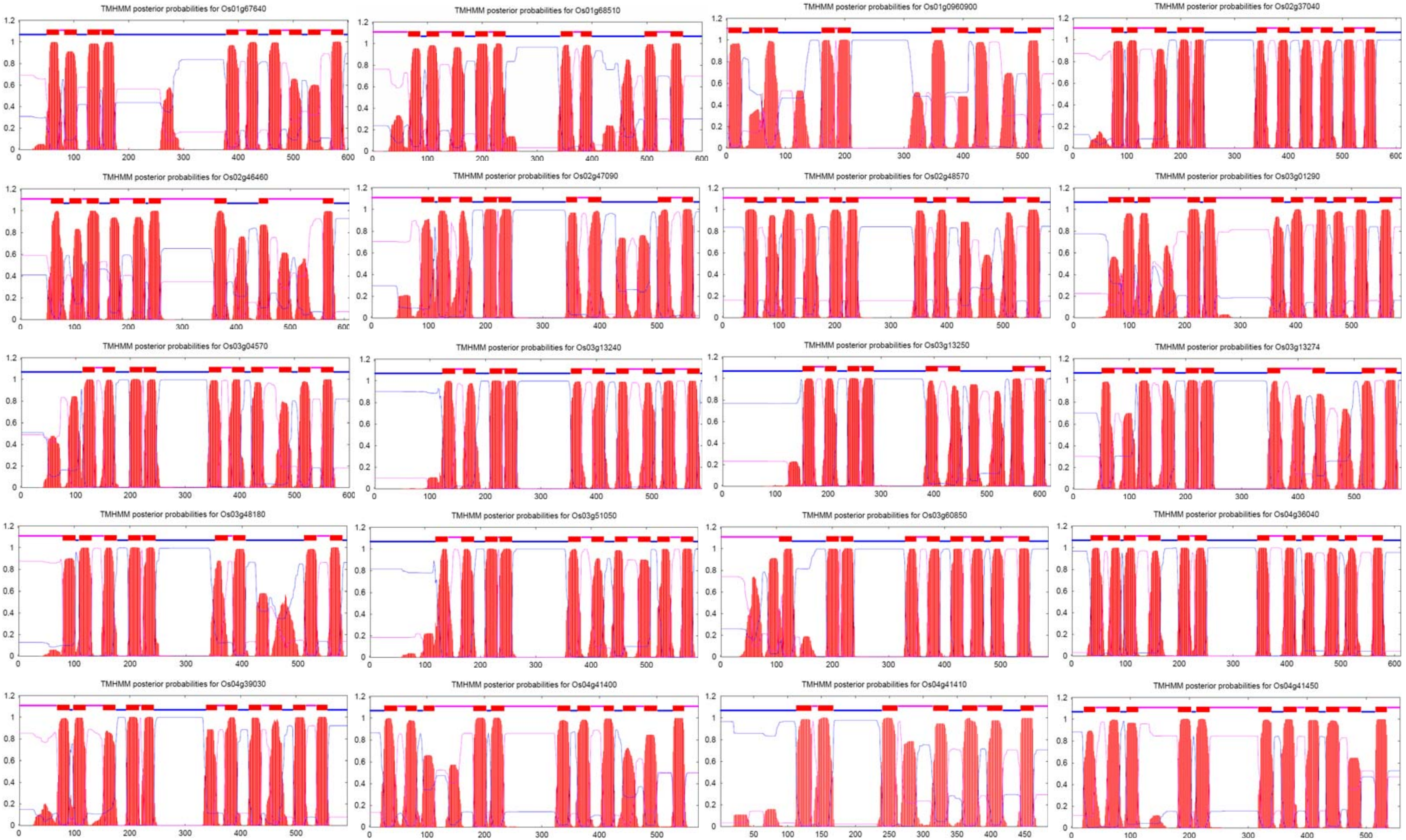
TMHMM posterior probabilities for Os01g67640
TMHMM posterior probabilities for Os01g68510
TMHMM posterior probabilities for Os01g0960900
TMHMM posterior probabilities for Os02g37040
TMHMM posterior probabilities for Os02g46460
TMHMM posterior probabilities for Os02g47090
TMHMM posterior probabilities for Os02g48570
TMHMM posterior probabilities for Os03g01290
TMHMM posterior probabilities for Os03g04570
TMHMM posterior probabilities for Os03g13240
TMHMM posterior probabilities for Os03g13250
TMHMM posterior probabilities for Os03g13274
TMHMM posterior probabilities for Os03g48180
TMHMM posterior probabilities for Os03g51050
TMHMM posterior probabilities for Os03g60850
TMHMM posterior probabilities for Os04g36040
TMHMM posterior probabilities for Os04g39030
TMHMM posterior probabilities for Os04g41400
TMHMM posterior probabilities for Os04g41410
TMHMM posterior probabilities for Os04g41450

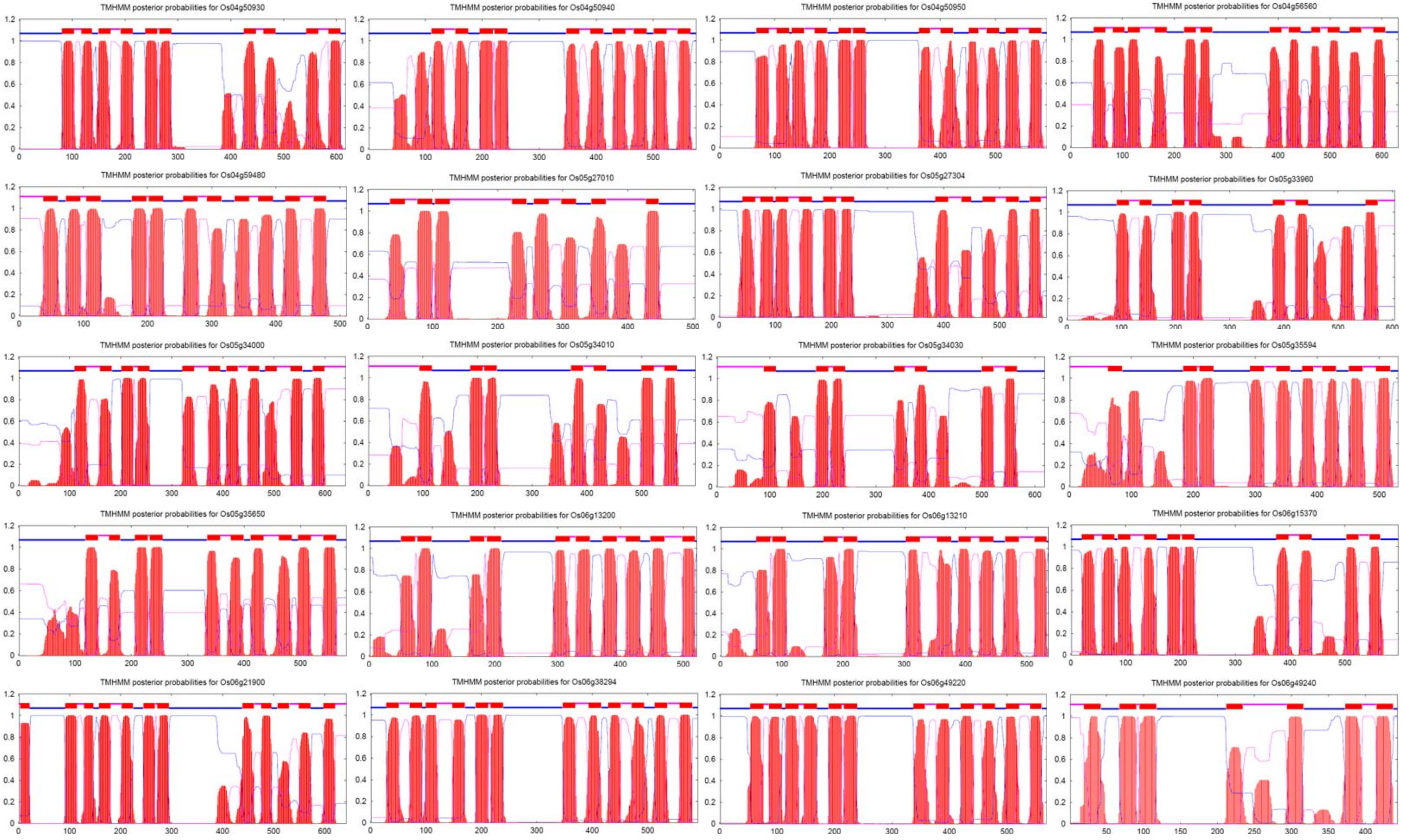
TMHMM posterior probabilities for Os04g50930
TMHMM posterior probabilities for Os04g50940
TMHMM posterior probabilities for Os04g50950
TMHMM posterior probabilities for Os04g56560
TMHMM posterior probabilities for Os04g59480
TMHMM posterior probabilities for Os05g27010
TMHMM posterior probabilities for Os05g27304
TMHMM posterior probabilities for Os05g33960
TMHMM posterior probabilities for Os05g34000
TMHMM posterior probabilities for Os05g34010
TMHMM posterior probabilities for Os05g34030
TMHMM posterior probabilities for Os05g35594
TMHMM posterior probabilities for Os05g35650
TMHMM posterior probabilities for Os06g13200
TMHMM posterior probabilities for Os06g13210
TMHMM posterior probabilities for Os06g15370
TMHMM posterior probabilities for Os06g21900
TMHMM posterior probabilities for Os06g38294
TMHMM posterior probabilities for Os06g49220
TMHMM posterior probabilities for Os06g49240

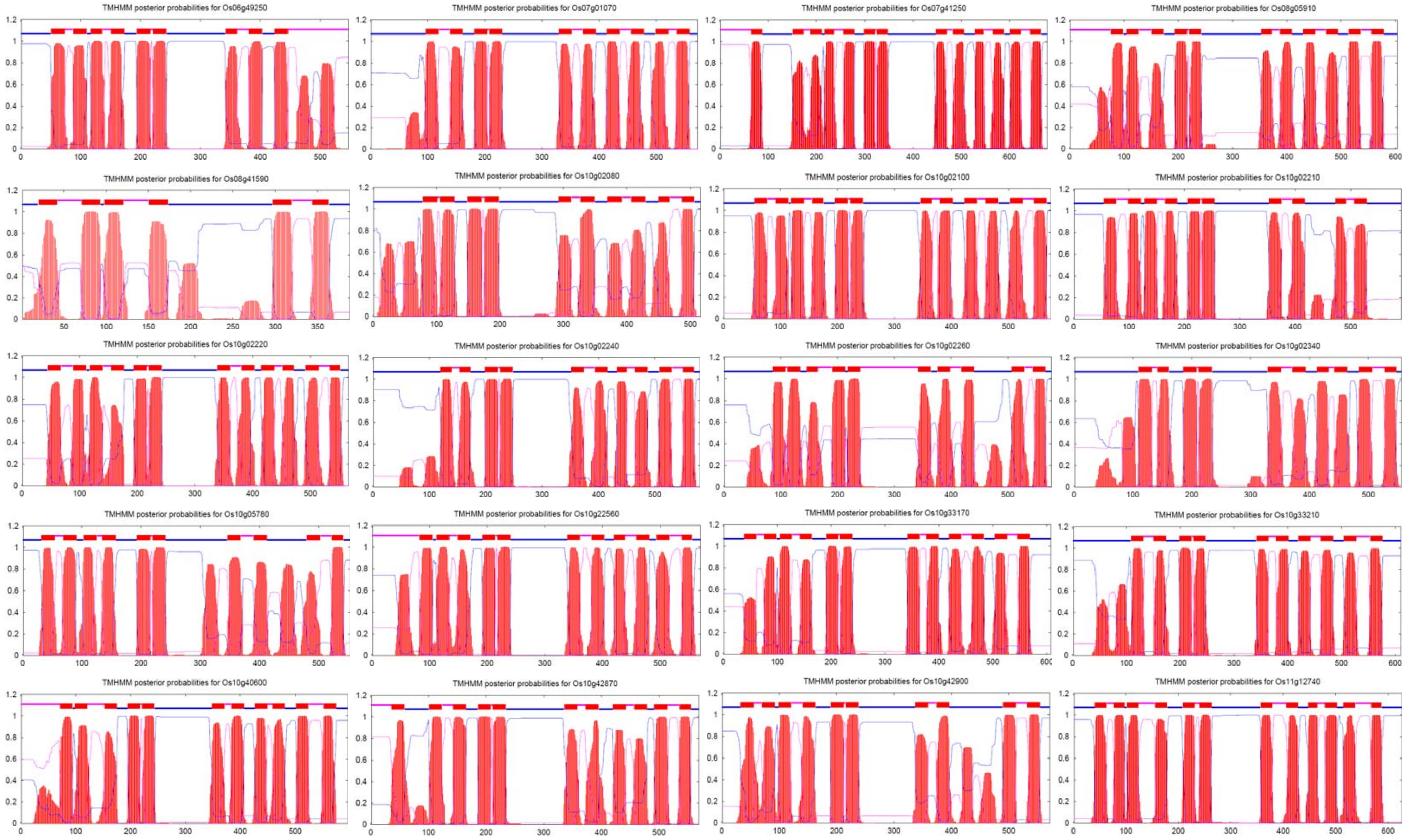
TMHMM posterior probabilities for Os06g49250
TMHMM posterior probabilities for Os07g01070
TMHMM posterior probabilities for Os07g41250
TMHMM posterior probabilities for Os08g05910
TMHMM posterior probabilities for Os08g41590
TMHMM posterior probabilities for Os10g02080
TMHMM posterior probabilities for Os10g02100
TMHMM posterior probabilities for Os10g02210
TMHMM posterior probabilities for Os10g02220
TMHMM posterior probabilities for Os10g02240
TMHMM posterior probabilities for Os10g02260
TMHMM posterior probabilities for Os10g02340
TMHMM posterior probabilities for Os10g05780
TMHMM posterior probabilities for Os10g22560
TMHMM posterior probabilities for Os10g33170
TMHMM posterior probabilities for Os10g33210
TMHMM posterior probabilities for Os10g40600
TMHMM posterior probabilities for Os10g42870
TMHMM posterior probabilities for Os10g42900
TMHMM posterior probabilities for Os11g12740

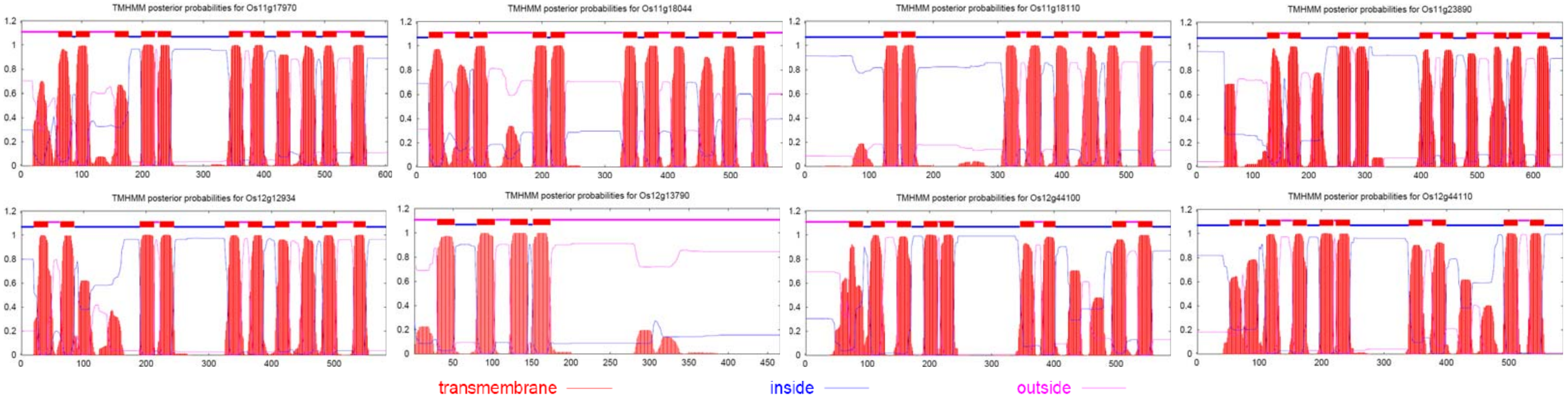

Gene	Length	Number of predicted TMs	Total prob of N-in	Positions of TM												
				TM1	TM2	TM3	TM4	TM5	TM6	TM7	TM8	TM9	TM10	TM11	TM12	TM13
Os01g01360	606	12	0.48475	78-100	127-149	182-201	221-243	248-270	366-388	401-423	438-460	467-484	489-511	523-545	571-590	
Os01g04950	580	10	0.7347	98-120	140-162	183-205	209-231	329-348	368-390	411-433	453-475	496-518	538-560			
Os01g37590	585	11	0.66957	78-100	105-127	147-169	189-211	216-238	342-362	377-399	420-439	459-481	501-523	538-557		
Os01g54515	528	11	0.0563	15-37	44-61	91-108	129-151	161-183	275-297	317-339	359-376	408-430	443-465	489-511		
Os01g55600	574	10	0.32555	62-84	91-113	139-161	182-204	214-236	366-388	413-435	448-470	485-507	531-553			
Os01g55610	590	9	0.18254	59-81	88-110	136-158	179-201	206-228	327-349	364-386	492-514	539-561				
Os01g65100	545	11	0.24697	74-96	109-131	146-168	198-220	224-246	302-324	344-366	387-409	429-451	463-485	505-527		
Os01g65110	548	9	0.90236	111-133	182-204	209-231	293-315	328-350	370-392	416-438	453-475	501-523				
Os01g65120	492	6	0.46869	25-44	51-73	338-357	378-400	415-437	458-477							
Os01g65140	341	4	0.7119	137-159	181-203	265-287	307-329									
Os01g65150	537	7	0.0693	62-84	96-118	215-237	335-357	377-399	423-445	506-525						
Os01g65169	541	7	0.0755	62-84	216-238	302-324	337-359	382-404	425-447	510-529						
Os01g65190	662	12	0.61767	29-51	66-88	95-117	145-162	189-211	216-238	429-446	461-483	504-526	548-570	582-604	631-650	
Os01g65200	361	7	0.43984	31-53	68-90	102-124	144-162	189-211	215-237	307-329						
Os01g65210	712	10	0.19766	73-95	107-129	139-156	214-236	304-326	338-360	380-402	427-449	464-486	507-529			

Os01g67630	557	12	0.78467	28-50	70-92	99-121	125-147	168-190	194-216	319-341	356-376	397-419	439-461	482-504	528-550
Os01g67640	599	10	0.30855	51-73	83-105	125-147	151-173	378-400	415-435	456-478	493-515	527-549	569-591		
Os01g68510	600	9	0.23718	65-87	99-121	145-167	188-210	220-242	343-365	380-399	496-518	543-565			
Os01g0960900	553	10	0.14234	4-26	39-61	65-87	161-183	188-210	347-369	391-408	421-443	463-485	509-531		
Os02g37040	609	11	0.12569	72-94	101-120	152-174	194-216	221-243	341-360	380-402	423-445	460-482	503-522	542-561	
Os02g46460	610	9	0.41021	57-79	91-113	123-145	166-183	209-231	238-260	360-382	442-459	561-580			
Os02g47090	574	9	0.29615	88-110	117-139	154-176	197-219	223-245	341-360	380-402	502-524	544-563			
Os02g48570	584	11	0.83776	39-61	74-96	106-128	149-171	191-213	220-242	342-364	376-398	418-440	501-523	543-565	
Os03g01290	589	11	0.77507	63-85	90-109	116-138	205-227	234-256	356-378	391-413	433-455	468-490	505-527	551-573	
Os03g04570	600	10	0.5104	113-135	150-172	199-221	225-247	344-366	386-408	421-443	472-494	507-529	549-571		
Os03g13240	591	10	0.90103	123-145	160-182	208-230	235-257	354-373	393-415	436-458	484-506	519-541	564-586		
Os03g13250	621	8	0.7683	152-174	194-216	237-259	264-286	385-407	427-449	549-571	591-610				
Os03g13274	584	10	0.69842	51-73	88-110	117-139	159-181	201-223	228-250	346-368	426-448	514-536	556-575		
Os03g48180	587	9	0.12652	80-102	109-131	154-176	197-219	223-245	352-374	384-406	511-533	557-579			
Os03g51050	593	10	0.81513	119-141	166-188	209-231	235-257	359-381	401-423	443-465	485-507	520-542	562-584		
Os03g60850	585	9	0.25882	105-127	189-211	215-237	330-349	369-391	411-433	448-470	483-505	532-551			
Os04g36040	611	12	0.96876	36-58	68-90	97-119	143-165	197-219	229-251	345-367	394-416	428-450	474-496	508-530	559-578
Os04g39030	592	11	0.14899	69-91	98-120	152-174	194-216	221-243	338-357	372-394	415-437	452-474	495-517	537-556	
Os04g41400	572	11	0.86436	26-48	63-82	94-113	181-203	212-234	327-349	362-384	408-425	441-463	478-500	528-547	
Os04g41410	484	7	0.96604	113-135	145-167	240-262	315-337	358-380	395-417	444-463					
Os04g41450	557	11	0.88332	21-40	60-82	94-113	181-203	212-234	317-339	360-382	397-419	432-454	469-491	516-535	
Os04g50930	617	10	0.99774	81-103	118-137	150-172	192-214	239-261	265-287	425-447	462-484	543-565	585-607		
Os04g50940	575	10	0.61615	111-133	153-175	196-218	222-244	348-370	390-412	429-451	466-488	501-523	543-565		
Os04g50950	593	12	0.89644	67-89	104-126	131-153	173-195	216-238	242-264	362-381	401-423	452-469	484-506	519-541	561-583
Os04g56560	631	12	0.60126	45-67	82-104	111-133	163-185	219-241	251-273	384-406	421-443	464-481	496-518	538-560	584-606
Os04g59480	510	11	0.09303	38-60	73-95	105-127	176-198	202-224	256-278	293-315	336-358	373-395	415-437	459-478	
Os05g27010	503	8	0.62977	35-57	77-99	104-126	222-244	256-278	298-320	344-366	428-447				
Os05g27304	583	11	0.98881	42-64	74-96	101-123	143-165	186-208	218-240	386-408	432-449	470-492	512-534	554-573	
Os05g33960	604	7	0.9621	92-114	134-156	194-216	226-248	380-402	422-444	551-573					
Os05g34000	640	11	0.60717	110-132	160-182	202-224	234-256	321-343	372-394	407-429	449-471	483-505	533-555	576-598	
Os05g34010	598	7	0.71709	94-116	187-209	213-235	371-393	413-435	500-522	542-564					
Os05g34030	618	7	0.35024	89-111	188-210	220-242	335-354	374-396	500-522	544-566					
Os05g35594	530	9	0.32169	63-85	184-206	210-232	292-314	334-356	376-393	408-430	451-473	495-517			
Os05g35650	581	10	0.33895	119-141	161-180	207-229	233-255	335-357	377-399	412-434	462-484	496-518	541-563		
Os06g13200	521	10	0.91679	51-73	77-99	161-183	188-210	297-319	329-351	372-394	409-431	448-470	499-518		

Os06g13210	535	10	0.77146	59-81	85-107	169-191	201-223	303-325	354-376	389-408	423-445	465-487	513-532			
Os06g15370	597	10	0.96753	20-42	57-79	86-108	134-156	177-199	203-225	375-397	417-439	503-525	545-564			
Os06g21900	642	12	0.07053	4-21	92-114	129-146	158-180	202-224	245-267	272-294	439-461	476-495	508-530	550-572	598-620	
Os06g38294	590	12	0.95129	29-51	71-93	100-119	148-170	190-212	217-239	347-369	394-416	429-451	471-493	513-535	558-580	
Os06g49220	569	12	0.99374	54-76	86-108	115-137	147-169	190-212	217-239	338-357	377-399	420-442	457-479	499-521	541-560	
Os06g49240	443	7	0.01241	20-42	68-90	95-117	212-234	294-316	373-395	415-437						
Os06g49250	547	9	0.97632	51-73	88-110	117-136	151-173	194-216	221-243	342-361	381-403	424-446				
Os07g01070	572	10	0.70794	97-119	139-161	182-204	208-230	330-352	372-394	414-436	456-478	499-521	536-558			
Os07g41250	678	13	0.02721	65-87	151-173	188-210	217-236	256-278	299-321	325-347	448-470	483-505	529-546	566-588	601-623	643-665
Os08g05910	603	11	0.58185	76-98	105-127	151-173	194-216	220-242	353-372	387-409	430-452	472-494	514-536	556-578		
Os08g41590	388	6	0.50673	20-42	71-93	98-120	151-173	297-319	344-363							
Os10g02080	516	10	0.81785	79-101	106-128	149-171	176-198	293-312	327-349	370-392	407-429	450-467	487-506			
Os10g02100	574	12	0.9503	55-77	92-114	119-141	156-175	196-218	223-245	346-365	380-402	423-445	460-482	503-525	545-567	
Os10g02210	591	10	0.96534	55-77	99-121	128-150	165-187	206-228	232-254	353-375	395-417	473-492	507-529			
Os10g02220	566	12	0.74717	45-67	89-111	118-140	155-177	194-216	220-242	339-361	381-403	415-437	452-471	492-514	534-551	
Os10g02240	580	10	0.90311	120-139	154-173	199-221	226-248	352-374	394-416	433-450	465-487	508-530	550-569			
Os10g02260	576	10	0.75826	86-108	112-134	146-165	191-213	218-240	342-364	376-398	418-440	507-529	544-566			
Os10g02340	556	10	0.63436	110-132	142-161	186-208	212-234	329-351	371-393	413-432	442-464	484-506	528-547			
Os10g05780	553	10	0.97619	32-54	69-91	103-125	135-157	193-215	219-241	346-368	390-412	480-502	522-541			
Os10g22560	570	11	0.74107	83-105	112-134	149-171	192-214	218-240	339-361	381-403	420-439	459-481	494-516	538-555		
Os10g33170	607	12	0.55925	39-61	76-98	105-127	142-164	191-213	217-239	342-364	377-399	419-441	461-483	504-526	546-568	
Os10g33210	610	10	0.88758	109-131	151-173	199-221	225-247	342-364	379-401	420-442	462-484	505-527	554-576			
Os10g40600	596	11	0.40364	72-94	99-121	153-175	195-217	221-243	349-371	384-406	427-449	459-481	502-524	552-574		
Os10g42870	569	11	0.18629	36-58	101-123	143-165	186-208	212-234	336-358	373-395	420-442	457-479	492-514	536-555		
Os10g42900	571	10	0.84612	33-55	70-92	99-121	141-163	190-212	216-238	337-359	374-396	490-512	532-554			
Os11g12740	626	12	0.95779	41-63	78-97	102-124	156-178	209-231	241-263	357-379	406-428	448-465	480-502	515-537	568-587	
Os11g17970	603	11	0.29801	62-84	91-113	155-177	198-220	225-247	343-365	378-400	421-443	463-485	497-519	543-565		
Os11g18044	583	11	0.68761	20-42	62-84	91-113	185-207	214-236	329-351	363-385	405-427	450-472	487-509	536-555		
Os11g18110	569	8	0.9132	123-145	150-172	313-335	345-367	388-410	432-454	467-489	522-541					
Os11g23890	652	10	0.95678	126-148	163-185	252-274	284-306	398-420	435-457	481-498	530-552	557-579	607-629			
Os12g12934	583	10	0.79897	21-43	63-85	190-212	222-244	326-348	363-385	406-428	448-470	482-504	531-550			
Os12g13790	466	4	0.27542	30-52	81-103	123-145	152-174									
Os12g44100	588	9	0.30491	71-93	106-128	148-170	191-213	217-239	346-368	383-402	494-516	536-558				
Os12g44110	587	10	0.81805	53-72	77-99	112-134	154-176	197-219	223-245	340-362	377-399	492-514	534-556			
