# Supplementary material for: Genomic survey, characterization and expression profile analysis of the peptide transporter family in rice (Oryza sativa L.)
Source: BMC Plant Biol. 2010 May 20;10:92. doi: 10.1186/1471-2229-10-92 (PMC3017762; doi:10.1186/1471-2229-10-92)

Additional file 4 – Phylogenetic relationship of rice and *Arabidopsis* PTR proteins

The unrooted tree was generated using ClustalX program by neighbor-joining method. Bootstrap values from 1000 replicates are indicated at each node. The orthologous proteins are marked with red circles and the AtPTR proteins are marked with blue triangles. The tree can be divided into five subfamilies from I to V.

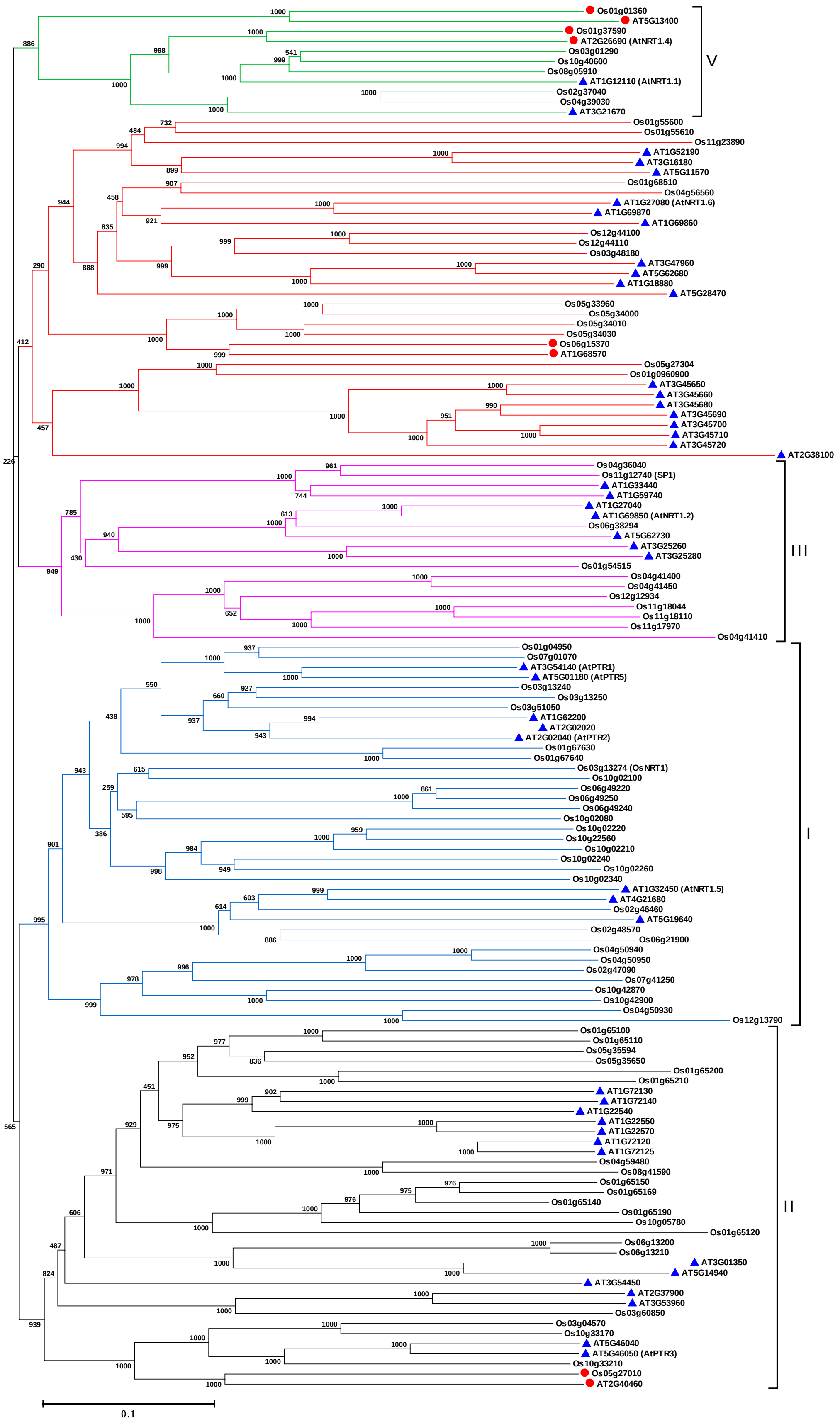

Supplement: Additional file 4 — Phylogenetic relationship of rice and Arabidopsis PTR proteins. The unrooted tree was generated using ClustalX program by neighbor-joining method. Bootstrap values from 1000 replicates are indicated at each node. The orthologous proteins are marked with red circles and the AtPTR proteins are marked with blue triangles. The tree can be divided into five subfamilies: I to V. [file 1471-2229-10-92-S4.PDF]
