# Supplementary material for: Genomic survey, characterization and expression profile analysis of the peptide transporter family in rice (Oryza sativa L.)
Source: BMC Plant Biol. 2010 May 20;10:92. doi: 10.1186/1471-2229-10-92 (PMC3017762; doi:10.1186/1471-2229-10-92)

Additional file 6 – Hierarchical cluster display of expression profile for 81 *OsPTR* genes with corresponding probes in ZS97

Color key represents log2 expression values. Developmental stages (Additional file 9) used for expression profiling are mentioned on bottom of each column. On the left side of expression map, cluster dendrogram is shown. On the right side, five groups have been marked with different rectangles for the genes showing different expression patterns.

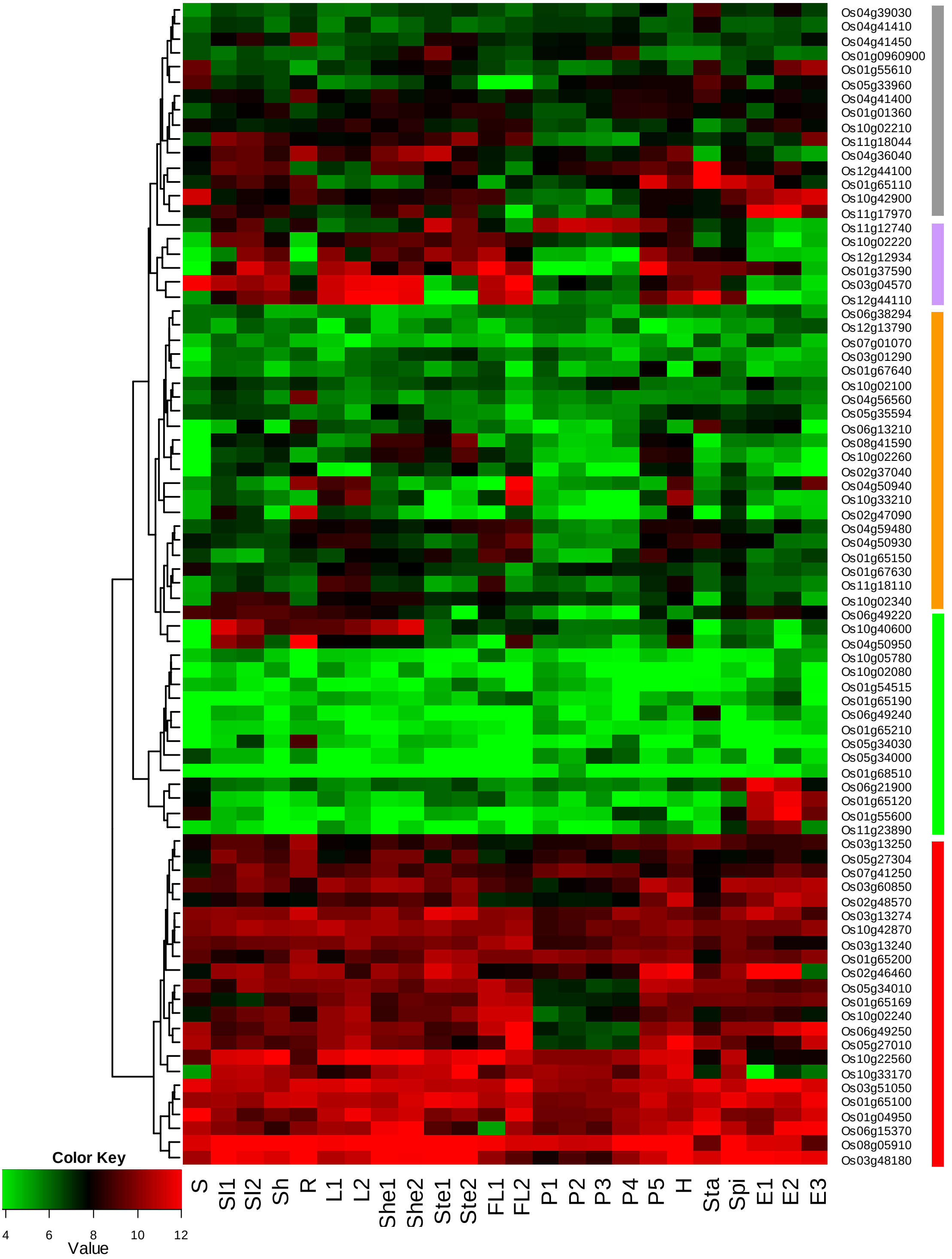

Supplement: Additional file 6 — Hierarchical cluster display of expression profile for 81 OsPTR genes with corresponding probes in ZS97. Color key represents log2 expression values. Developmental stages (Additional file 9) used for expression profiling are mentioned on bottom of each column. On the left side of expression map, cluster dendrogram is shown. On the right side, five groups have been marked with different rectangles for the genes showing different expression patterns. [file 1471-2229-10-92-S6.PDF]
