# Supplementary material for: Genomic survey, characterization and expression profile analysis of the peptide transporter family in rice (Oryza sativa L.)
Source: BMC Plant Biol. 2010 May 20;10:92. doi: 10.1186/1471-2229-10-92 (PMC3017762; doi:10.1186/1471-2229-10-92)
Supplement: Additional file 7 — Hierarchical cluster display of expression profile for 50 AtPTR genes with corresponding probes. Color key at the left represents log2 expression values. Developmental stages (list in the table below) used for expression profiling are mentioned on bottom of each column. On the left side of expression map, cluster dendrogram is shown. [file 1471-2229-10-92-S7.PDF]

Additional file 7 – Hierarchical cluster display of expression profile for 50 *AtPTR* genes with corresponding probes

Color key represents log2 expression values. Developmental stages (list in the table below) used for expression profiling are mentioned on bottom of each column. On the left side of expression map, cluster dendrogram is shown.

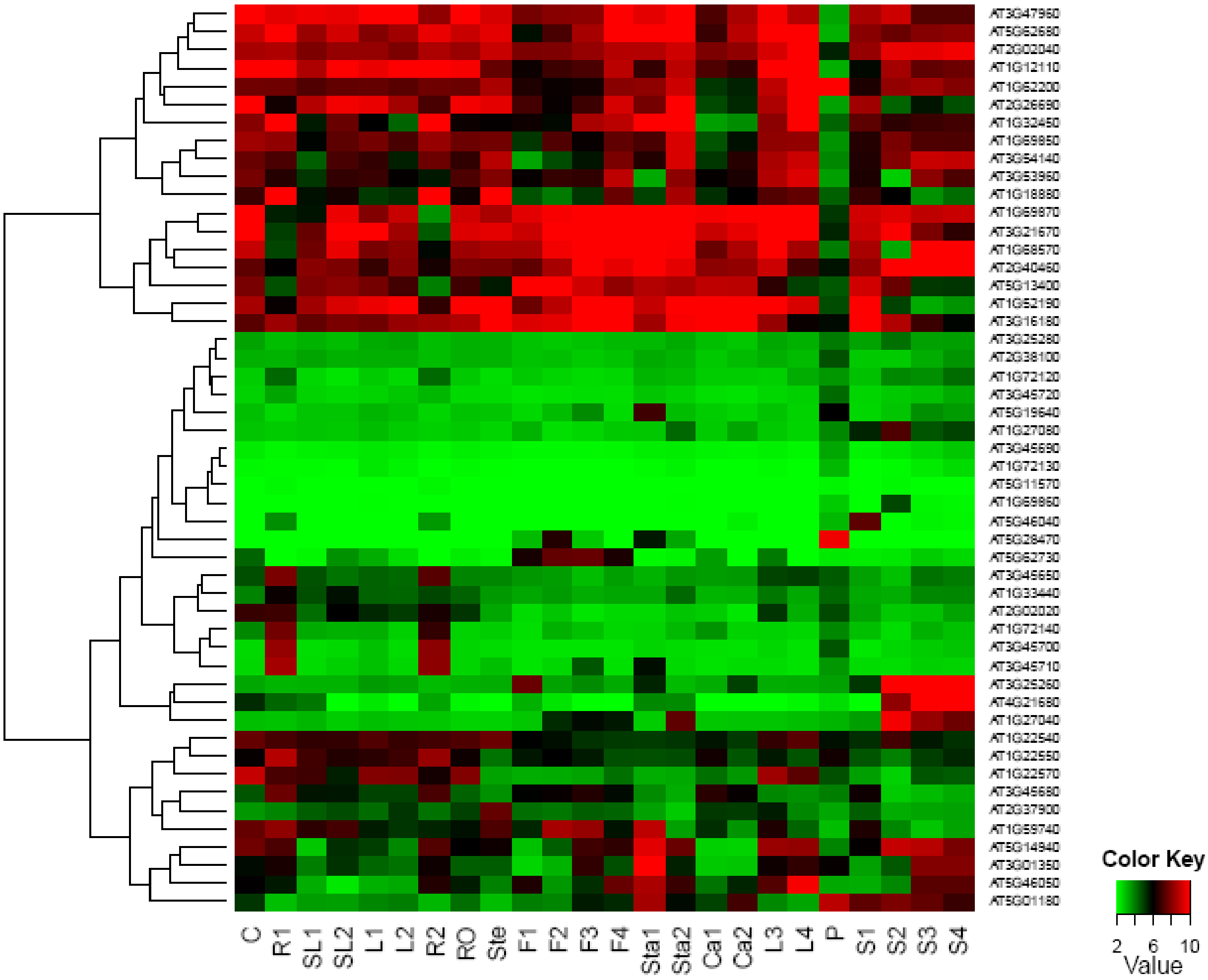

Sample ID	Tissue	Age	Abbreviation
1	cotyledons	7 days	C
2	roots	7 days	R1
3	shoot apex, vegetative + young leaves	7 days	SL1
4	seedling, green parts	7 days	SL2
5	leaves 1 + 2	7 days	L1
6	rosette leaf # 12	17 days	L2
7	roots	17 days	R2
8	developmental drift, entire rosette after	21 days	RO
9	stem, 2nd internode	21+ days	Ste
10	flowers stage 9	21+ days	F1
11	flowers stage 10/11	21+ days	F2
12	flowers stage 12	21+ days	F3
13	flowers stage 15	21+ days	F4
14	flowers stage 12, stamens	21+ days	Sta1
15	flowers stage 15, stamens	21+ days	Sta2
16	flowers stage 12, carpels	21+ days	Ca1
17	flowers stage 15, carpels	21+ days	Ca2
18	cauline leaves	21+ days	L3
19	senescing leaves	35 days	L4
20	mature pollen	6 wk	P
21	siliques, w/ seeds stage 3; mid globular to	8 wk	S1
22	seeds, stage 6, w/o siliques; mid to late	8 wk	S2
23	seeds, stage 9, w/o siliques; curled cotyledons	8 wk	S3
24	seeds, stage 10, w/o siliques; green cotyledons	8 wk	S4
