# Supplementary material for: Genomic survey, characterization and expression profile analysis of the peptide transporter family in rice (Oryza sativa L.)
Source: BMC Plant Biol. 2010 May 20;10:92. doi: 10.1186/1471-2229-10-92 (PMC3017762; doi:10.1186/1471-2229-10-92)
Supplement: Additional file 9 — Detailed information of rice samples used in microarray analysis [file 1471-2229-10-92-S9.PDF]

Additional file 9 – Detailed information of rice samples used in microarray analysis

Sample NO.	Developmental stage	Tissue	Abbreviation
1	72 hours after dry seed absorbed water	Seed	S
2	3 days after sowing	Seedling 1	Sl1
3	Trefoil stage	Seedling 2	Sl2
4	Seedling with 2 tillers	Shoot	Sh
5	Seedling with 2 tillers	Root	R
6	Young panicle: secondary branch primordium differentiation	Leaf 1	L1
7	Young panicle: meiosis stage	Leaf 2	L2
8	Young panicle: secondary branch primordium differentiation	Sheath 1	She1
9	Young panicle: meiosis stage	Sheath 2	She2
10	5 days before heading	Stem 1	Ste1
11	Heading stage	Stem 2	Ste2
12	5 days before heading	Flag Leaf 1	FL1
13	14 days after flowering	Flag Leaf 2	FL2
14	Young panicle: secondary branch primordium differentiation	Panicle 1	P1
15	Young panicle: pistil/stamen primordium differentiation	Panicle 2	P2
16	Young panicle: pollen-mother cell formation	Panicle 3	P3
17	Young panicle: meiosis stage	Panicle 4	P4
18	Heading stage	Panicle 5	P5
19	1 day before flowering	Hull	H
20	1 day before flowering	Stamen	Sta
21	3 day after flowering	Spikelet	Spi
22	7 days after pollination	Endosperm 1	E1
23	14 days after pollination	Endosperm 2	E2
24	21 days after pollination	Endosperm 3	E3
